# A numerical analysis of the water-based ternary hybrid nanofluid between two rotating disks subject to thermal radiation and homogeneous–heterogeneous reactions

**DOI:** 10.1186/s11671-026-04460-6

**Published:** 2026-02-14

**Authors:** Ebrahem A. Algehyne, Marwa M. Alzubaidi, Rabab Alzahrani, Anwar Saeed, Gabriella Bognár

**Affiliations:** 1 Department of Mathematics, Faculty of Science, University of Tabuk, P.O. Box 741, 71491 Tabuk, Saudi Arabia; 2 Department of Mathematics, College of Science and Humanities in Al-Kharj, Prince Sattam bin Abdulaziz University, 11942 Al-Kharj, Saudi Arabia; 3 Department of Mathematics, Abdul Wali Khan University, Mardan, Khyber Pakhtunkhwa 23200 Pakistan; 4 Institute of Machine and Product Design, University of Miskolc, Miskolc-Egyetemváros, 3515 Hungary

**Keywords:** Nanofluid, Ternary hybrid nanofluid, MHD, Homogeneous-heterogeneous chemical reaction, Rotating disks

## Abstract

This work involves detailed numerical research on tri-hybrid nanofluid flow that takes place between two gyrating disks with integrated effects of the magnetic field, Joule heating, thermal radiation, as well as, homogeneous-heterogeneous reactions. The main equations have solved through bvp4c approach in dimensionless from. It has found in this work that the stretching factor at the bottom disk increases the axial flow, and the radial flow exhibits a twofold action. Axial and tangential velocities are directly proportional to Reynolds number but inversely proportional to the effects of the magnet whereas radial velocity is a mixed proportion. The rotational factor of tangential flow increases. Thermal profiles increase as Eckert, Reynolds, magnetic and radiation parameters increase. The concentration undergoes a decreasing trend when the homogeneous factors, heterogeneous factors and Schmidt numbers are increased and an increasing trend when Reynolds number is increased. There is great congruence in comparative results and Nusselt and Sherwood number are measured in tables and are used to determine the heat and mass transfer. This study contains useful information on how to optimize reactive thermal systems using rotating geometries and multimodal nanofluids, and can find its applications in energy systems and chemical reactors as well as innovative cooling technologies.

## Introduction

Nanofluid flow presents the motion of pure fluid with nanoparticles mixed in it, in extremely small sizes, usually between 1 and 100 nm. Such nanoparticles suggestively augment the thermal and physical features of the host fluid as examined by Choi [1]. Shaaban et al. [2] investigated nanofluid flow on a surface while considering the role of microorganisms. Their study examined how microbes interact with the fluid, influence its movement, and affect overall flow behavior by altering stability, transport characteristics, and boundary layer dynamics. Anwar et al. [3] discussed magnetized nanofluid flow in a Darcy space using thermally radiative thermal source effects. The addition of nanoparticles causes a change in viscosity, density and heat capacity of the fluid that causes a better performance of the fluid in terms of heat transfer during flow as analyzed by Yasmine et al. [4]. The nanofluids have response variations when transported by channels, pipes, rotating systems, etc. when compared to the ordinary fluids, because of the enhanced thermal diffusion and Brownian movement of the particles. Flow Nanofluid flow has also been widely used in engineering, such as cooling systems, microfluidics, biomedical equipment, heat exchangers and renewable energy technology [5, 6]. Since nanofluids are more efficient and fast in heat transfer, they contribute to the better functioning of equipment, the decrease in the use of energy, and the possibility to make more compact and reliable thermal management systems. Tri-hybrid nanofluid studies enhanced distinct type of nanofluid which comprise of three divers types of nanoparticles blended together in one base fluid. As compared to traditional nanofluids or even hybrid nanofluids where one or two types of nanoparticles are employed only, tri-hybrid nanofluids possess a high thermal conductivity, stability, and rheological characteristics as a result of the synergistic action of three different types of nanoparticles. Abbas et al. [7] examined thermo-bioconvective trihybrid nanofluid flow with effects of microorganisms by implementing neural network approach. Farkhad et al. [8] discussed the augmentation of thermal flow for thermo-bioconvectve flow for trihybrid nanofluid with effects of microbes. Abbas et al. [9] utilized an ANN technique with Bayesian regularization to study the convective flow behavior of a trihybrid nanofluid, incorporating local thermal non-equilibrium effects and thermal radiation, and showed that the approach accurately captured complex heat transfer phenomena. Researchers investigate tri-hybrid nanofluids because of their ability to outperform traditional fluids in systems where efficient thermal management is essential, such as in electronic cooling, solar collectors, chemical reactors, and gyrating disk systems. Abbas et al. [10] examined the radiative trihybrid nanofluid flow by usning Cattaneo-Christove flux model with effects of microorganisms. The tri-hybrid nanofluids and nanofluids also produce a substantial change in the velocity and temperature of a flowing system. The ability of nanoparticles to conduct heat higher raises the thermal conductivity that facilitates the transfer of heat faster to different parts of the fluid as well as improving the temperature distribution.

Magnetohydrodynamic (MHD) flow application is the movement of electrically conductive magnetic-affected fluids. MHD-induced Lorentz force is in the middle of controlling the behavior of the fluid by resisting its movement and altering the transport of momentum and energy. MHD flow theory is a theory that incorporates fluid mechanics and electromagnetism. MHD fluid flow has been of great interest in numerous applications of engineering and industry systems due to its good flow control capacity. Anwar et al. [11] investigated the thermal transference features for magnetized nanofluid flow with varying thermal radiative effects. Khan et al. [12] investigated magnetized nanofluid flow over irregular three-dimensional surfaces, examining the influence of different slip effects on velocity and thermal behavior, highlighting how magnetic fields and surface irregularities impact flow characteristics and heat transfer performance. MHD is used in cooling systems of nuclear reactors, electromagnetic pumps, crystal growth processes, MHD generators, metallurgical processes, and biomedical engineering, such as blood flow control under magnetic fields [13, 14]. Khan et al. [15] discussed the ANN approach for modeling and solution of magnetized stagnant point flow of Ree-Eyring fluid thermally convective elongated sheet. In recent years, MHD flow has also become important in renewable energy systems and advanced thermal management technologies. In addition, MHD effects are particularly applicable in high temperature conditions where ionization rises electrical conductivity. Cham et al. [16] considered computationally the magnetized fluid flow in a permeable conduit. MHD fluid flow offers an efficient means of managing fluid movement and heat transfer and, therefore, it has been a major field of research in contemporary fluid dynamics as made apparent by Ali et al. [17]. To enable researchers to find more serious real-life examples, MHD flow is commonly investigated with other physical influences like the thermal radiation, chemical reactions, porous media, rotation, and nanofluid suspension [18, 19]. Guedri et al. [20] considered the flow of an EMHD nanofluid over a stretchable surface in terms of thermal dissipation and entropy generation in their article, showing the effect of electromagnetic fields and surface stretching on energy loss, heat transfer as well as the overall thermodynamic efficiency of the nanofluid system. Homogenous-heterogeneous chemical reactions Fluid flow Fluid flow in which the reaction involves a homogeneous-heterogeneous reaction is a large field of study in chemical, mechanical, and environmental engineering. In these systems the chemical reaction can take place in the bulk of the fluid (homogeneous reactions) as well as on the surface or interface of solid catalysts or particles of fluid (heterogeneous reactions). Homogeneous reactions are in which the reactants are completely dissolved or suspended in the fluid and the rate of reaction is related to concentration, temperature and fluid dynamics. Heterogeneous reactions on the other hand happen on the interface between the fluid and a solid surface and may depend on the properties of the surface, adsorption effects, and the catalyst activity. Hussain Shah et al. [21] employed such reactions to examine the thermal transportation of nanofluid flow. Mass, momentum and energy transport in the fluid is complicating since these reactions occur simultaneously with each other as reaction rates are linked to flow patterns and heat transfer as Jubair et al. [22] and Roja et al. [23] argue. One example of homogeneous-heterogeneous reactions is common in industrial processes and includes catalyst reactors, pollutant removal in water treatment process, combustion, pharmaceutical production, and biochemical reactions [24, 25]. Fatima et al. [26] studied the heterogeneous- homogeneous chemical reaction effects on heat transference augmentation of fluid flow. They may increase the overall reaction rate with respect to bulk fluid dynamics as well as surface phenomenon. Also, chemical reactions occur, which changes temperature profiles, has an impact on stability of flows, and development of boundary layers. According to Jena et al. [27], velocity and thermal panels are greatly affected by the processes of the reaction in fluid flow where the chemical reactions are of homogeneous-heterogeneous type. Exothermic reactions raise the temperature of fluid and therefore thicken thermal boundary layer whereas endothermic reactions may reduce the temperature and make the layer thin as shown by Khan et al. [28]. Naveed et al. [29] pointed out that heterogeneous/homogeneous fluid flow with chemical reactions are therefore critical in the regulation of the reaction processes, enhancement of energy efficiency and reduction of the environmental impact. Bashir et al. [30] scrutinized the impression of the magnetic field on the flow of nanofluid using the impact of CNTs.

Flow of fluids in rotating disks is a classical and significant problem of fluid mechanics, which has extensive uses in engineering and industrial systems. Such kind of flow usually takes place between a pair of parallel disks where one or both of the disks turns around a common axis. The rotation creates complicated patterns of flow. Radial acceleration acting on the fluid particles is experienced as the disks rotate and the fluid is shifted away to the disk edges as opposed to the centre of the disks. This radial flow movement is counteracted by an axial inflow creating a three dimensional swirling pattern of flow. Uddin et al. [31] investigated fluid flow using two gyrating disks flux model of Cattaneo-Christov. Alharbi et al. [32] examined the growth in thermal transference of fluid flow in tow disks that had dissipative and thermally radiative effects. Lone et al. [33] found that the flow behavior is highly dependent on the parameters like the rotation speed of the disks, fluid viscosity, and the disks moving in same or opposite directions. Some of the common practical applications and uses of rotating disk flows include gas turbine, computer hard drives, centrifugal pumps, chemical reactors, rotating heat exchanger, and aerospace systems [34–36]. Ahmad et al. [37] modeled the electro-chemical radiative fluid flow between two gyrating disk by calculating the effects of electrochemical processes and thermal radiation that affect the flow behavior, velocity distribution, and heat transfer properties in the rotating disk system. Li et al. [38] investigated that rotating disks were common in thermal engineering to promote heat transfer because of augmented mixing and turbulence caused by rotation. Khan et al. [39] examined the significances of the nanofluid flow utilizing gyrating disks and the effects of microorganisms. In the cases of heat transfer, where the heat transfer is associated with viscous dissipation and rotational effects, the heat distribution will be affected, and the thermal boundary layer may be altered considerably. The rotating disk flow of the fluids is critical to the optimization of the system performance, better cooling, less mechanical losses, and stability of the rotating machinery used in high-tech engineering processes [40].

In light of the above-reviewed literature, the present analysis considers the water-based trihybrid nanofluid flow comprising of MgO, CoFe_2_O_4_ and TiO_2_ nanoparticles between two rotating disks. The impacts of Joule heating, magnetic field, thermal radiation and homogeneous-heterogeneous chemical reactions are taken into consideration. Due to the presence such effects, the physical model captures complex interaction between thermal transport and chemical reactive species within the nanofluid system. This analysis is presented to respond the undermentioned questions:

- How do the axial, tangential and radial velocity components of the ternary hybrid nanofluid respond to variations in external forces?
- How do the temperature distribution and thermal boundary layers behave against the governing factors?
- How do the heterogeneous/homogeneous chemical reactions affect the concentration profile and mass transport behavior of the reactive species in the nanofluid?
- How do the engineering quantities of interest vary against the key governing parameters?

## Problem formulation

**Fig. 1:**
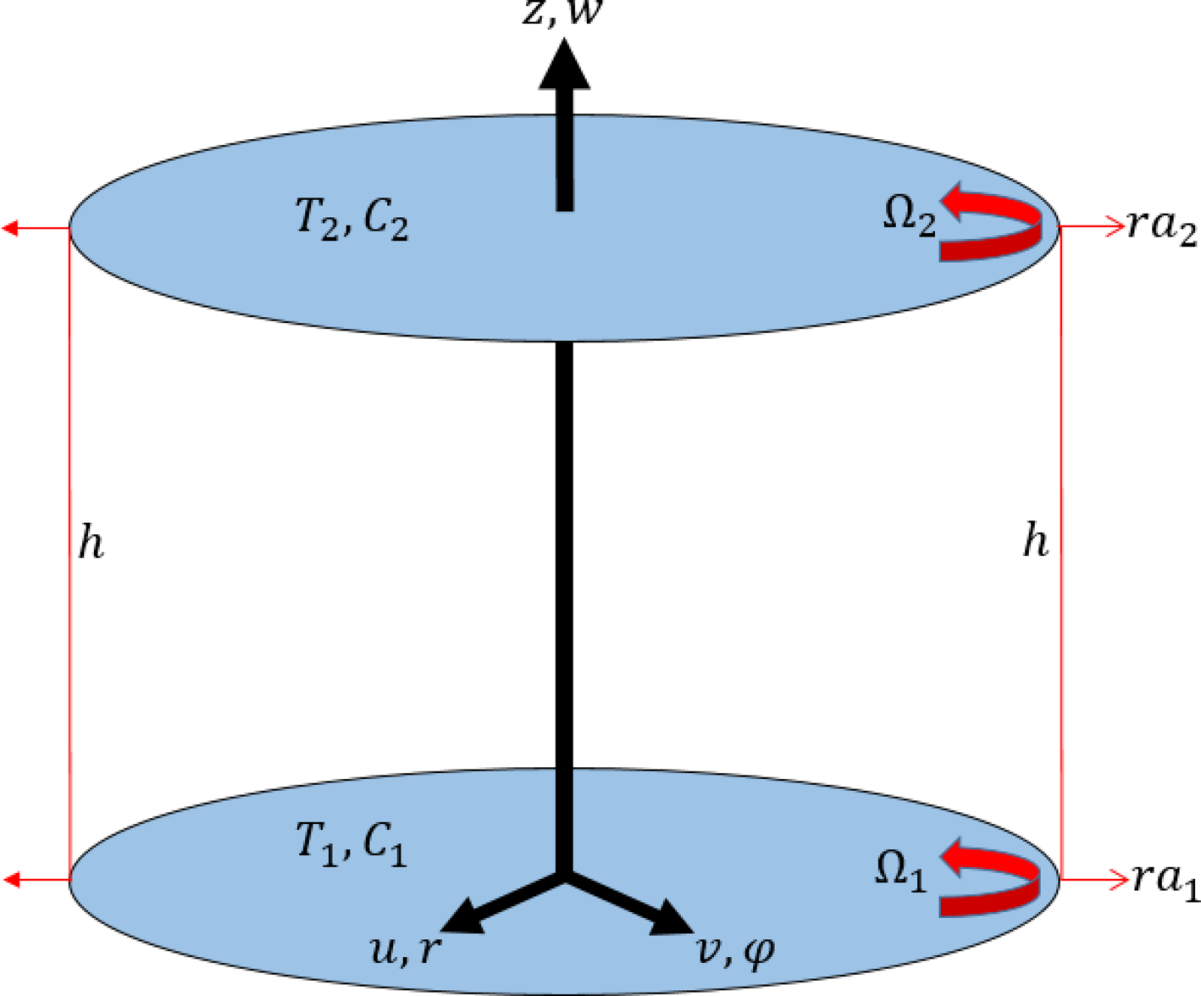
Geometrical veiw of flow problem

Assume the time-independent, viscous and incompressible flow of ternary hybrid nanofluid containing *MgO*, *CoFe*_*2*_*O*_*4*_ and *TiO*_*2*_ nanoparticles among two rotating disks. The two disks which are uniformly spaced with a distance *h*, rotates about their own axes of rotations. $$\Omega_{1}$$ and $$\Omega_{2}$$ are the velocities of lower and upper disks respectively as illustarted in Fig. 1. Additionally, $$a_{1} r$$ and $$a_{2} r$$ are the rates with which lower and upper disks are stretched, where $$a_{1}$$ and $$a_{2}$$ are elongating constants. $$B_{0}$$ is intensity of magnetic filed employed in z-direction. Lower and upper disks have temperatures and concentration as $$T_{1}$$,$$C_{1}$$ and $$T_{2}$$$$C_{2}$$, respectively. The impacts of Joule heating, and thermal radiation are used in the wrok. For modification in mass transfer rate, homogeneous-heterogeneous chemical reactions are also used. The isothermal cubic autocatalysis for a homogeneous reaction is characterized by [41]:

$$ A + 2B \to 3B,\,\,\,\,\,{\mathrm{Rate}} = k_{c} ab^{2} , $$

While for hetrogeneous reaction we have:

$$ A \to B,\,\,\,\,\,{\mathrm{Rate}} = k_{s} a, $$

The homogeneous and heterogeneous reactions occurring in a boundary layer motion are related by the aforementioned mathematical expressions. Reactant $A$ has $a_{0}$ as fixed concentration, in these isothermal reactions that occur away from the plate when auto-catalyst $B$ is not present in the external flow.

Using above mentioned assumptions, the main equations can be written as [42–44]:

1 $$ \frac{\partial u}{{\partial r}} + \frac{u}{r} + \frac{\partial w}{{\partial z}} = 0, $$

2 $$ u\frac{\partial u}{{\partial r}} + w\frac{\partial u}{{\partial z}} - \frac{{v^{2} }}{r} = - \frac{1}{{\rho_{hnf} }}\frac{\partial p}{{\partial r}} + \nu_{Thnf} \left( {\frac{{\partial^{2} u}}{{\partial r^{2} }} + \frac{1}{r}\frac{\partial u}{{\partial r}} + \frac{{\partial^{2} u}}{{\partial z^{2} }} - \frac{u}{{r^{2} }}} \right) - \frac{{\sigma_{Thnf} }}{{\rho_{Thnf} }}B_{0}^{2} u, $$

3 $$ u\frac{\partial v}{{\partial r}} + \frac{u\,v}{r} + w\frac{\partial v}{{\partial z}} = \nu_{Thnf} \left( {\frac{{\partial^{2} v}}{{\partial z^{2} }} + \frac{{\partial^{2} v}}{{\partial r^{2} }} - \frac{v}{{r^{2} }} + \frac{1}{r}\frac{\partial v}{{\partial r}}} \right) - \frac{{\sigma_{Thnf} }}{{\rho_{Thnf} }}B_{0}^{2} \,v, $$

4 $$ u\frac{\partial w}{{\partial r}} + w\frac{\partial w}{{\partial z}} = - \frac{1}{{\rho_{hnf} }}\frac{\partial p}{{\partial z}} + \nu_{hnf} \left( {\frac{{\partial^{2} w}}{{\partial r^{2} }} + \frac{1}{R}\frac{\partial w}{{\partial r}} + \frac{{\partial^{2} w}}{{\partial z^{2} }}} \right), $$

5 $$ \begin{aligned} \left( {\rho c_{p} } \right)_{hnf} \left( {u\frac{\partial T}{{\partial r}} + w\frac{\partial T}{{\partial z}}} \right) & = \left( {k_{hnf} + \frac{{16\sigma^{*} T_{2}^{3} }}{{3k^{**} }}} \right) + \left( {\frac{1}{r}\frac{\partial T}{{\partial r}} + \frac{{\partial^{2} T}}{{\partial r^{2} }} + \frac{{\partial^{2} T}}{{\partial z^{2} }}} \right) \\ & + \sigma_{hnf} B_{0}^{2} (u^{2} + v^{2} ), \\ \end{aligned} $$

6 $$ u\frac{\partial a}{{\partial r}} + w\frac{\partial a}{{\partial z}} = D_{a} \left( {\frac{{\partial^{2} a}}{{\partial r^{2} }} + \frac{1}{r}\frac{\partial a}{{\partial r}} + \frac{{\partial^{2} a}}{{\partial z^{2} }}} \right) - k_{c} ab^{2} , $$

7 $$ u\frac{\partial b}{{\partial r}} + w\frac{\partial b}{{\partial z}} = D_{b} \left( {\frac{{\partial^{2} b}}{{\partial r^{2} }} + \frac{1}{r}\frac{\partial b}{{\partial r}} + \frac{{\partial^{2} b}}{{\partial z^{2} }}} \right) + k_{c} ab^{2} , $$

with boundary conditions [41–44]:

8 $$ \begin{aligned} & u = a_{1} r,\,v = \Omega_{1} r,\,w = 0,\,T = T_{1} ,\,D_{a} \frac{\partial a}{{\partial z}} = k_{s} a,\,D_{b} \frac{\partial b}{{\partial z}} = - k_{s} a,\,\,{\mathrm{at}}\,\,z = 0 \\ & u = a_{2} r,\,v = \Omega_{2} r,\,w = 0,\,T = T_{2} ,\,a \to a_{0} ,\,b \to 0\,\,{\mathrm{at}}\,\,z = h. \\ \end{aligned} $$

The thermophysical features of MgO, CoFe_2_O_4_, TiO_2_ and H_2_O are re listed in Table 1.

**Table 1:** Thermophysical properties of the solid nanoparticles and base fluid [45]

	MgO	CoFe_2_O_4_	TiO_2_	H_2_O
$\rho$	3560	4907	4250	997.1
$C_{p}$	955	700	686.2	4179
$k$	45	3.7	8.9538	0.613
$\sigma$	1.42 × 10^–3^	5.51 × 10^9^	2.38 × 10^6^	0.05

The thermophysical relations for mono, hybrid and ternary hybrid nanofluids are defined in Tables 2, 3 and 4.

**Table 2:** Thermophysical relations of the nanofluid [46]

Properties	Relation
Density	$\rho_{nf} = \phi \,\,\rho_{1} + \rho_{f} \,\left( {1 - \phi } \right)$
Viscosity	$\mu_{nf} = \frac{{\mu_{f} }}{{\left( {1 - \phi_{1} } \right)^{2.5} }}$
Specific Heat Capacity	$\left( {\rho C_{p} } \right)_{nf} = \left( {1 - \phi_{1} } \right)\left( {\rho C_{p} } \right)_{f} + \phi_{1} \left( {\rho C_{p} } \right)_{1}$
Thermal Conductivity	$k_{nf} = \left( {\frac{{k_{1} - 2\,\phi_{1} \left( {k_{f} - k_{1} } \right) + 2\,k_{f} }}{{k_{1} + \phi_{1} \,\,\left( {k_{f} - k_{1} } \right) + 2\,k_{f} }}} \right)k_{f}$
Electrical Conductivity	$\sigma_{nf} = \left( {\frac{{\sigma_{1} + 2\sigma_{f} - 2\phi_{1} \left( {\sigma_{f} - \sigma_{1} } \right)}}{{\sigma_{1} + 2\sigma_{f} + \phi_{1} \left( {\sigma_{f} - \sigma_{1} } \right)}}} \right)\sigma_{f}$

**Table 3:** Thermophysical relations of the hybrid nanofluid [45, 46]

Properties	Relation
Density	$\rho_{hnf} = \left( {\left( {1 - \phi_{1} } \right)\rho_{f} + \phi_{1} \rho_{1} } \right)\left( {1 - \phi_{2} } \right)$ $+ \phi_{2} \rho_{2}$
Viscosity	$\mu_{hnf} = \frac{{\mu_{f} }}{{\left( {1 - \phi_{2} } \right)^{2.5} \left( {1 - \phi_{1} } \right)^{2.5} }}$
Specific Heat Capacity	$\left( {\rho C_{p} } \right)_{hnf} = \left( {\left( {\rho C_{p} } \right)\left( {1 - \phi_{1} } \right)_{f} + \left( {\rho C_{p} } \right)_{1} \phi_{1} } \right)$ $\left( {1 - \phi_{2} } \right) + \phi_{2} \left( {\rho C_{p} } \right)_{2}$
Thermal Conductivity	$k_{hnf} = \left( {\frac{{k_{2} - 2\,\phi_{2} \left( {k_{nf} - k_{2} } \right)\, + 2k_{nf} }}{{k_{2} + \phi_{2} \left( {k_{nf} - k_{2} } \right) + 2k_{nf} }}} \right)k_{nf}$
Electrical Conductivity	$\sigma_{hnf} = \left( {\frac{{\sigma_{2} - 2\,\phi_{2} \left( {\sigma_{nf} - \sigma_{2} } \right) + 2\sigma_{nf} }}{{\sigma_{2} + \phi_{2} \left( {\sigma_{n\,f} - \sigma_{2} } \right) + 2\sigma_{nf} }}} \right)\sigma_{nf}$

**Table 4:** Thermophysical relations of the ternary hybrid nanofluid [45, 46]

Properties	Relation
Density	$\rho _{{Thnf}} = \left( {1 - \phi _{1} } \right)\left[ {\left( {1 - \phi _{2} } \right)\left\{ {\left( {1 - \phi _{3} } \right) + } \right.} \right.$ $+\left. {\phi _{3} \frac{{\rho _{3} }}{{\rho _{f} }}} \right\} + \left. { \phi _{2} \frac{{\rho _{2} }}{{\rho _{f} }}} \right] + \phi _{1} \frac{{\rho _{1} }}{{\rho _{f} }}$
Viscosity	$\mu_{Thnf} = \frac{{\mu_{f} }}{{\left( {1 - \phi_{1} } \right)^{2.5} \left( {1 - \phi_{2} } \right)^{2.5} \left( {1 - \phi_{3} } \right)^{2.5} }}$
Specific Heat Capacity (*Cp*)	$\left( {\rho C_{p} } \right)_{{Thnf}} = \left( {1 - \phi _{1} } \right)\left[ {\left( {1 - \phi _{2} } \right)\left\{ {\left( {1 - \phi _{3} } \right)} \right.} \right.$ $+ \left. {\phi _{3} \frac{{\left( {\rho C_{p} } \right)_{3} }}{{\left( {\rho C_{p} } \right)_{f} }}} \right\} + \left. {\phi _{2} \frac{{\left( {\rho C_{p} } \right)_{2} }}{{\left( {\rho C_{p} } \right)_{f} }}} \right] + \phi _{1} \frac{{\left( {\rho C_{p} } \right)_{1} }}{{\left( {\rho C_{p} } \right)_{f} }}$
Thermal Conductivity	$k_{Thnf} = k_{hnf} \left[ {\frac{{k_{3} + 23_{hnf} - 2\phi_{3} \left( {k_{hnf} - k_{3} } \right)}}{{k_{3} + 2k_{hnf} + \phi_{3} \left( {k_{hnf} - k_{3} } \right)}}} \right]$
Electrical Conductivity	$\sigma_{Thnf} = \left( {\frac{{\sigma_{3} + 2\sigma_{hnf} - 2\phi_{3} \left( {\sigma_{hnf} - \sigma_{3} } \right)}}{{\sigma_{3} + 2\sigma_{hnf} + \phi_{3} \left( {\sigma_{hnf} - \sigma_{3} } \right)}}} \right)\sigma_{hnf}$

Variable of similarity are given as [41, 46]:

9 $$ \begin{aligned} & u = r\Omega_{1} F^{\prime}\left( \eta \right),\,v = rG\left( \eta \right)\Omega_{1} ,\,w = - 2h\Omega_{1} F\left( \eta \right),\,\theta = \frac{{T - T_{2} }}{{T_{1} - T_{2} }}, \\ & p = \rho_{f} \nu_{f} \Omega_{1} \left( {P\left( \eta \right) + \frac{{r^{2} }}{{2h^{2} }}\varepsilon } \right),\,\eta = \frac{z}{h},\,a = a_{0} g\left( \eta \right),\,b = a_{0} j\left( \eta \right), \\ \end{aligned} $$

Using Eq. (9), we have:

10 $$ F^{iv} \left( \eta \right) + l_{1} l_{2} {\mathrm{Re}} \left( {2F\left( \eta \right)F^{\prime\prime\prime}\left( \eta \right) + 2G\left( \eta \right)G^{\prime}\left( \eta \right) - \frac{{l_{3} }}{{l_{2} }}MF^{\prime\prime}\left( \eta \right)} \right) = 0, $$

11 $$ G^{\prime\prime}\left( \eta \right) + l_{1} l_{2} {\mathrm{Re}} \left( {2F\left( \eta \right)G^{\prime}\left( \eta \right) - 2F^{\prime}\left( \eta \right)G\left( \eta \right) - M\frac{{l_{3} }}{{l_{2} }}G\left( \eta \right)} \right) = 0, $$

12 $$ \frac{1}{{\Pr l_{5} }}\left( {l_{4} + Rd} \right)\theta^{\prime\prime}\left( \eta \right) + 2{\mathrm{Re}} F\left( \eta \right)\theta^{\prime}\left( \eta \right) + {\mathrm{Re}} EcM\frac{{l_{3} }}{{l_{5} }}\left( {F^{{\prime}{2}} \left( \eta \right) + G^{2} \left( \eta \right)} \right) = 0, $$

13 $$ P^{\prime}\left( \eta \right) + 4l_{2} {\mathrm{Re}} F\left( \eta \right)F^{\prime}\left( \eta \right) + \frac{2}{{l_{1} }}F^{\prime\prime}\left( \eta \right) = 0, $$

14 $$ \frac{1}{Re}\frac{1}{Sc}g^{\prime\prime}\left( \eta \right) + 2F\left( \eta \right)g^{\prime}\left( \eta \right) - k_{1} g\left( \eta \right)j^{2} \left( \eta \right) = 0, $$

15 $$ \frac{1}{Re}\frac{\delta }{Sc}j^{\prime\prime}\left( \eta \right) + 2F\left( \eta \right)j^{\prime}\left( \eta \right) - k_{1} g\left( \eta \right)j^{2} \left( \eta \right) = 0. $$

The related conditions are:

16 $$ \begin{aligned} & F\left( 0 \right) = 0,F^{\prime}\left( 0 \right) = \alpha_{1} ,F\left( 1 \right) = 0,F^{\prime}\left( 1 \right) = \alpha_{2} , \\ & G\left( 0 \right) = 1,G\left( 1 \right) = \Omega , \\ & \theta \left( 0 \right) = 1,\theta \left( 1 \right) = 0, \\ & P\left( 0 \right) = 0, \\ & g^{\prime}\left( 0 \right) = K_{2} g\left( 0 \right),g\left( 1 \right) = 1, \\ & \delta j^{\prime}\left( 0 \right) = K_{2} g\left( 0 \right),j\left( 1 \right) = 0. \\ \end{aligned} $$

Here $$l_{1} = \frac{{\mu_{Thnf} }}{{\mu_{f} }}$$, $$l_{2} = \frac{{\rho_{Thnf} }}{{\rho_{f} }}$$, $$l_{3} = \frac{{\sigma_{Thnf} }}{{\sigma_{f} }}$$, $$l_{4} = \frac{{\left( {\rho C_{p} } \right)_{Thnf} }}{{\left( {\rho C_{p} } \right)_{f} }}$$ and $$l_{5} = \frac{{k_{Thnf} }}{{k_{f} }}$$.

17 $$ \begin{aligned} & M = \frac{{\sigma_{f} B_{0}^{2} }}{{\rho_{f} \Omega_{1} }},{\mathrm{Re}} = \frac{{\Omega_{1} h^{2} }}{{\nu_{f} }},\delta = \frac{{D_{C} }}{{D_{B} }},Sc = \frac{{\nu_{f} }}{{D_{B} }},k_{1} = \frac{{k_{c} b_{0}^{2} }}{{\Omega_{1} }},k_{2} = \frac{{k_{c} h}}{{D_{B} }}, \\ & \alpha_{1} = \frac{{a_{1} }}{{\Omega_{1} }},Ec = \frac{{r^{2} \Omega_{1}^{2} }}{{\left( {T_{1} - T_{2} } \right)\left( {c_{p} } \right)_{f} }},\alpha_{2} = \frac{{a_{2} }}{{\Omega_{1} }},\Omega = \frac{{\Omega_{2} }}{{\Omega_{1} }},\Pr = \frac{{\left( {\rho C_{p} } \right)_{f} \nu_{f} }}{{k_{f} }}. \\ \end{aligned} $$

In Eq. (17), $\alpha_{1}$ and $\alpha_{2}$ are the stretching factor at lower and upper surface, ${\mathrm{Re}}$ is Reynolds number, $\delta$ is diffusion coefficient ratio, $\Omega$ is ratio factor, $\Pr$ is Prandtl number, $Ec$ is Eckert number, $M$ is magnetic factor, $Sc$ is Schmidt number, $k_{1}$ and $k_{2}$ are homogeneous and heterogeneous reaction parameter, respectively.

Given that the coefficient $A$ and $B$ are similar in size, it follows that the diffusion coefficients $D_{a}$ and $D_{b}$ are equivalent, indicating that $\delta = 1$. Accordingly, a significant relationship between $g\left( \eta \right)$ and $j\left( \eta \right)$ may be expressed as [47].

18 $$ g\left( \eta \right) + j\left( \eta \right) = 1, $$

From expression (14) and (15), we obtain:

19 $$ \frac{1}{{\mathrm{Re}}}\frac{1}{Sc}g^{\prime\prime}\left( \eta \right) + 2F\left( \eta \right)g^{\prime}\left( \eta \right) - K_{1} g\left( \eta \right)\left( {1 - g\left( \eta \right)} \right)^{2} = 0, $$

20 $$ g^{\prime}\left( 0 \right)\,\,\, = K_{2} \,\,g\left( 0 \right),\,\,\,\,\,g\left( 1 \right) = 1. $$

Further

21 $$ P\left( \eta \right) = - 2\left\{ {F^{\prime}\left( \eta \right) - F^{\prime}\left( 0 \right) + {\mathrm{Re}} \frac{{l_{2} }}{{l_{1} }}F^{2} \left( \eta \right)} \right\}. $$

The Nusselt number of lower and upper disks are

22 $$ \dot{N}u_{x1} = \left. {\frac{{hq_{w} }}{{k_{f} \left( {T_{1} - T_{2} } \right)}}} \right|_{z = 0} ,\quad \dot{N}u_{x2} = \left. {\frac{{hq_{w} }}{{k_{f} \left( {T_{1} - T_{2} } \right)}}} \right|_{z = h} , $$

where

23 $$ q_{w} = - k_{Thnf} \frac{\partial T}{{\partial z}} + q_{r} , $$

and $$q_{r} = - \frac{{16\sigma^{*} T_{2}^{3} }}{{3k^{**} }}\frac{\partial T}{{\partial z}}$$.

24 $$ {\mathrm{Therefore}},\quad Nu_{1} = \left( {\frac{{k_{Thnf} }}{{k_{f} }} + Rd} \right)\theta^{\prime}\left( 0 \right),Nu_{2} = \left( {\frac{{k_{Thnf} }}{{k_{f} }} + Rd} \right)\theta^{\prime}\left( 1 \right). $$

The Sherwood number of lower and upper disks are

25 $$ Sh_{x1} = \left. {\frac{{q_{m} }}{{D_{b} \left( {C_{1} - C_{2} } \right)}}} \right|_{z = 0} ,\quad Sh_{x2} = \left. {\frac{{q_{m} }}{{D_{b} \left( {C_{1} - C_{2} } \right)}}} \right|_{z = h} , $$

26 $$ {\mathrm{Where}}\quad q_{m} = - D_{b} \frac{\partial C}{{\partial z}}. $$

27 $$ Sh_{1} = - g^{\prime}\left( 0 \right),\quad Sh_{2} = - g^{\prime}\left( 1 \right) $$

## Solution methodology

For solution of above modeled equations we shall employ bvp4c approach. It is a numerical approach that is effectively used to evaluate the nonlinear differential equations. It exhibits rapid convergence and is used in many engineering and applied physics problems. We employed the bvp4c with relative error tolerance of 10^–6^ to ensure high numerical accuracy. For utilization, this technique necessitates the conversion of higher order ODEs to 1st order ODEs as explained below for current problem.

To perform this method, the following steps are followed:

Step 1: Convert the higher-order ODEs into first-order ODEs.

Step 2: The boundary conditions are formulated in terms of first-order ODEs and defined initial and final points for computational domain.

Step 3: Use a uniform mesh to initiate the numerical procedure.

Step 4: Apply the convergence criteria to solve the system of first-order ODEs.

Step 5: Post-processing the results for physical interpretation.

Therefore, we consider:

28 $$ \begin{aligned} & y_{1} = f,y_{2} = f^{\prime},y_{3} = f^{\prime\prime},y_{4} = f^{\prime\prime\prime},y^{\prime}_{4} = f^{iv} \\ & y_{5} = G,y_{6} = G^{\prime},y^{\prime}_{6} = G^{\prime\prime}, \\ & y_{7} = \theta ,y_{8} = \theta^{\prime},y^{\prime}_{8} = \theta^{\prime\prime},y_{9} = P,y^{\prime}_{9} = P^{\prime} \\ & y_{10} = g,y_{11} = g^{\prime},y^{\prime}_{11} = g^{\prime\prime} \\ \end{aligned} $$

Using Eq. (28) in Eqs. (10–15) with boundary conditions in Eq. (16), the following first order equations are obtained:

29 $$ y^{\prime}_{4} = - \left\{ {l_{1} l_{2} {\mathrm{Re}} \left( {2y_{1} y_{4} + 2y_{5} y_{6} - \frac{{l_{3} }}{{l_{2} }}My_{3} } \right)} \right\} $$

30 $$ y^{\prime}_{6} = - \left\{ {l_{1} l_{2} {\mathrm{Re}} \left( {2y_{1} y_{6} - 2y_{2} y_{5} - M\frac{{l_{3} }}{{l_{2} }}y_{5} } \right)} \right\} $$

31 $$ y^{\prime}_{8} = - \frac{{\Pr l_{5} }}{{\left( {l_{4} + Rd} \right)}}\left\{ {2{\mathrm{Re}} y_{1} y_{8} + {\mathrm{Re}} EcM\frac{{l_{3} }}{{l_{5} }}\left( {y_{2}^{2} + y_{5}^{2} } \right)} \right\} $$

32 $$ y^{\prime}_{9} = - \left\{ {4l_{2} {\mathrm{Re}} y_{1} y_{2} + \frac{2}{{l_{1} }}y_{3} } \right\} $$

33 $$ y^{\prime}_{11} = - {\mathrm{Re}} Sc\left\{ {2y_{1} y_{11} - K_{1} y_{10} \left( {1 - y_{10} } \right)^{2} } \right\} $$

The associated transformed boundary conditionsa are:

34 $$ \begin{aligned} & y_{1a} - 0,y_{2a} - \alpha_{1} ,y_{1b} - 0, \\ & y_{2b} - \alpha_{2} ,y_{5a} - 1,y_{5b} - \Omega , \\ & y_{7a} - 1,\,\;y_{7b} - 0,y_{9a} - 0, \\ & y_{11a} - K_{2} y_{10a} ,y_{10b} - 1. \\ \end{aligned} $$

## Validation

For the urpose of validation this work has matched in Table 5 with already established works of Hosseinzadeh et al. [48] and Gul et al. [46] for deviations in rotational number $\left( \Omega \right)$ while fixing all other factors as $M = \alpha_{1} = \alpha_{2} = \phi_{1} = \phi_{2} = \phi_{3} = 0$ and ${\mathrm{Re}} = 1.0$. Excellent agreement among the findings verifies the accuracy of the present work.

**Table 5:** Comparison of the present work with the published work of Hosseinzadeh et al. [48] and Gul et al. [46] when $M = \alpha_{1} = \alpha_{2} = \phi_{1} = \phi_{2} = \phi_{3} = 0$ and ${\mathrm{Re}} = 1.0$

$\Omega$	Hosseinzadeh et al. [48]		Gul et al. [46]		Present	
	$F^{\prime\prime}\left( 0 \right)$	$- G^{\prime}\left( 0 \right)$	$F^{\prime\prime}\left( 0 \right)$	$- G^{\prime}\left( 0 \right)$	$F^{\prime\prime}\left( 0 \right)$	$- G^{\prime}\left( 0 \right)$
− 1.0	0.06666313	2.0009523	0.06659549	2.00095025	0.06665861	2.0009490
− 0.8	0.08394206	1.8025943	0.08385833	1.80258332	0.08385519	1.8025848
− 0.3	0.10395088	1.3044324	0.10384713	1.30441473	0.10389274	1.3044405
0.0	0.09997221	1.0042857	0.09987239	1.00426903	0.09989046	1.0042683
0.5	0.06663419	0.5026190	0.06656769	0.50260830	0.06659638	0.5026072

## Discussion of results

In this study, a numerical research on tri-hybrid nanofluid flow of two gyrating disks is done with effects of mixed convection, Joule heating, and homogeneous-heterogeneous chemical reaction. The physical model models the complicated interplay of forces caused by rotation, thermal transport and reactive species in the nanofluid. The dimension-free coupled leading equations are resolved effectively in the MATLAB inbuilt solver bvp4c that provides high precision and numerical stability. The impression of diverse factors on velocity and temperature panels have expounded in subsequent paragraphs. The default values of the embedded factors are defined as $M = 0.1$, ${\mathrm{Re}} = 0.1$, $Sc = 2.0$, $k_{1} = 0.1$, $k_{2} = 0.1$, $\alpha_{1} = 0.1$, $Ec = 0.1$, $\alpha_{2} = 0.1$ and $\Omega = 0.5$.

### Velocity profiles

**Fig. 2:**
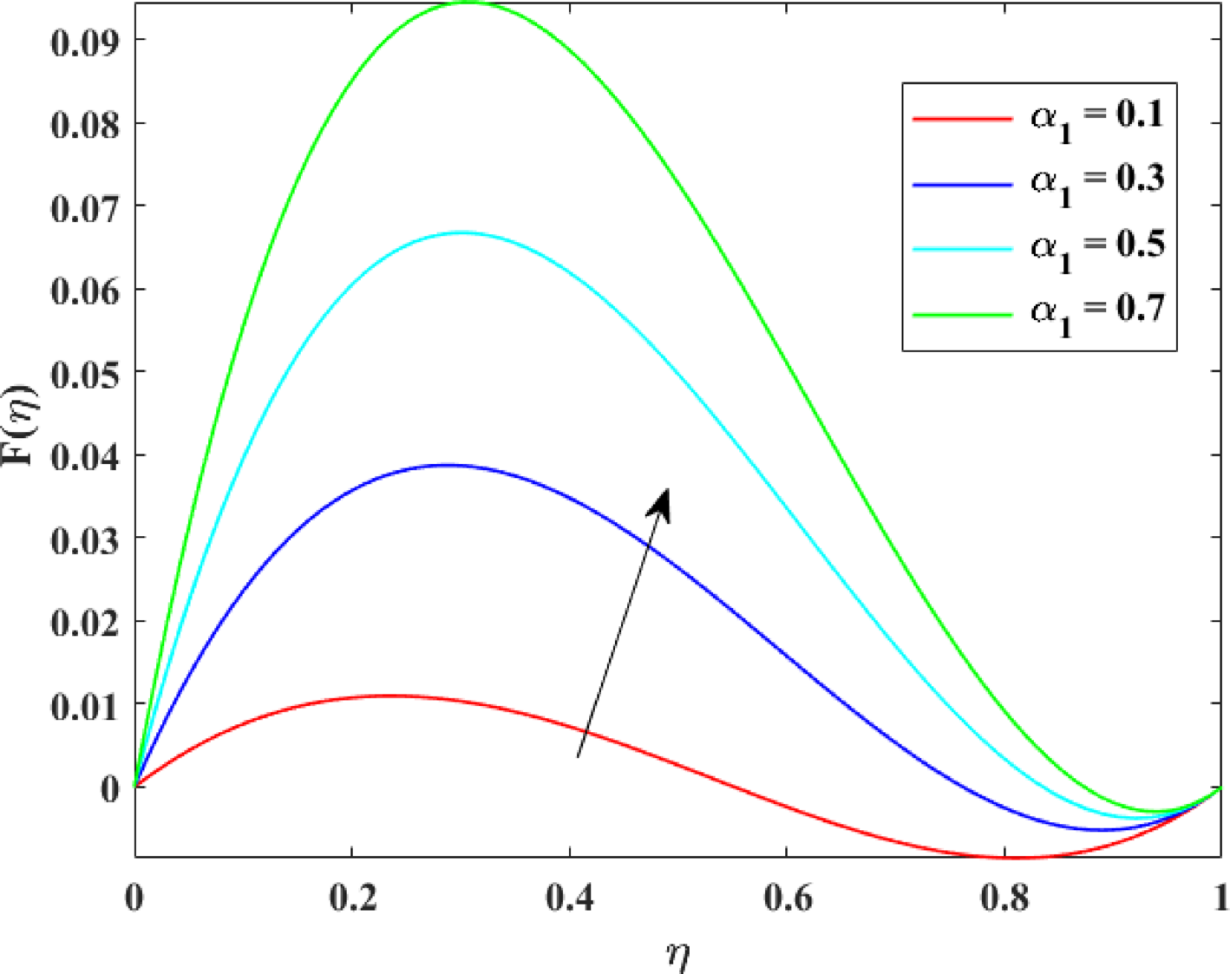
Impact of $\alpha_{1}$ on $F\left( \eta \right)$

**Fig. 3:**
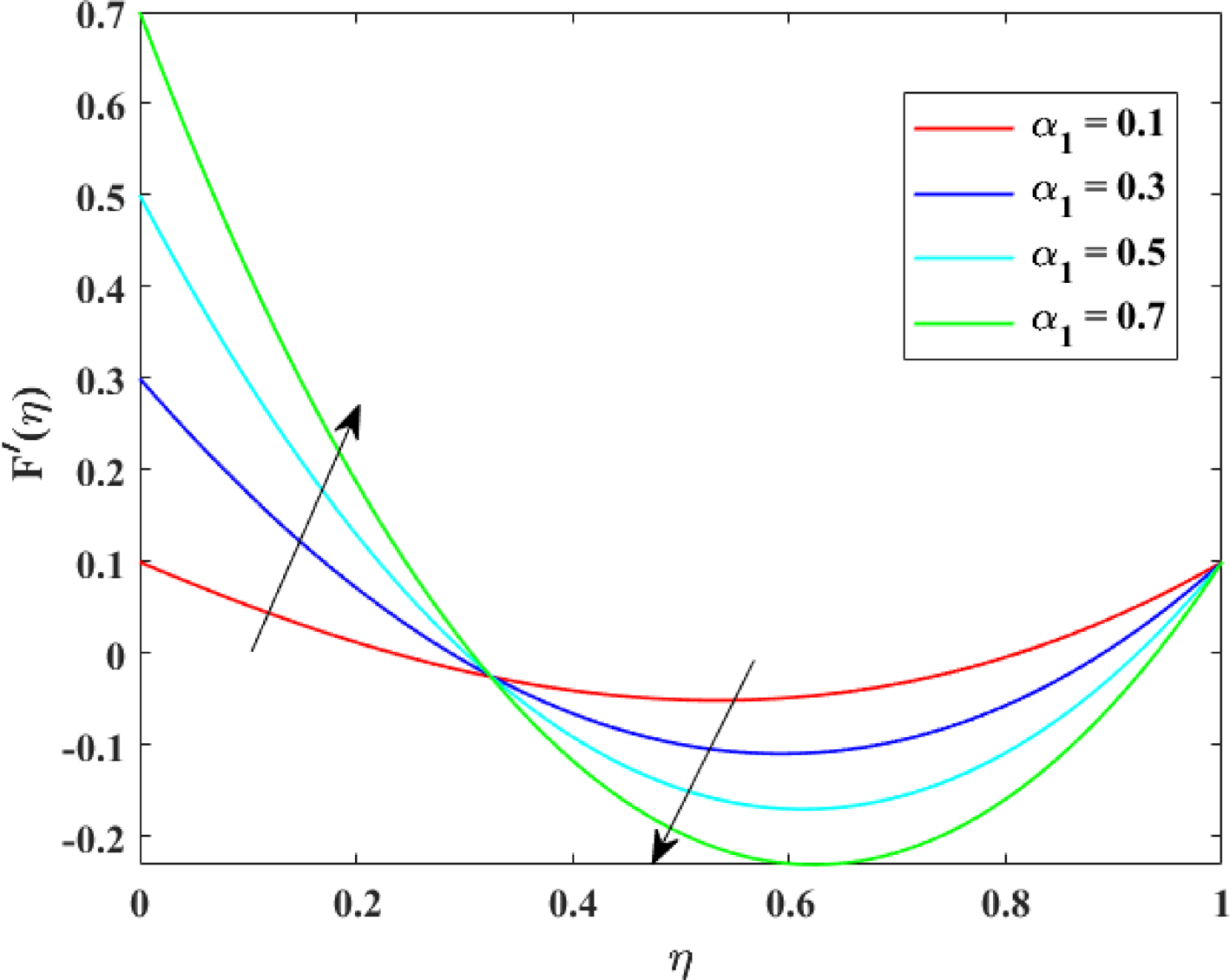
Impact of $\alpha_{1}$ on $F^{\prime}\left( \eta \right)$

**Fig. 4:**
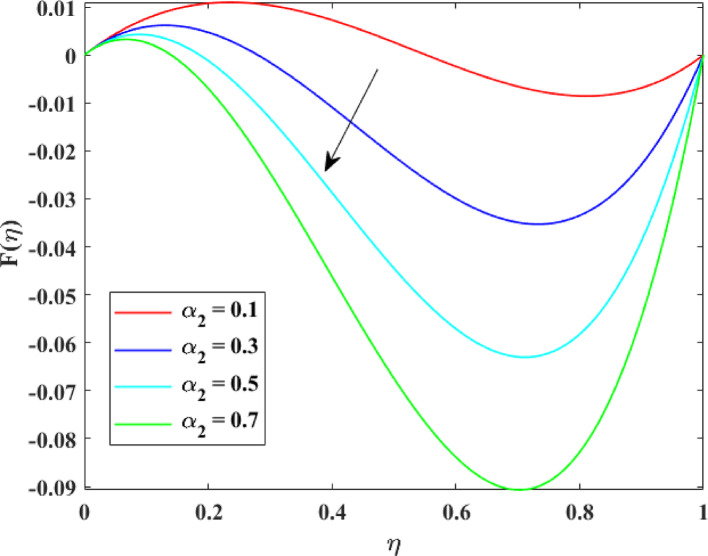
Impact of $\alpha_{2}$ on $F\left( \eta \right)$

**Fig. 5:**
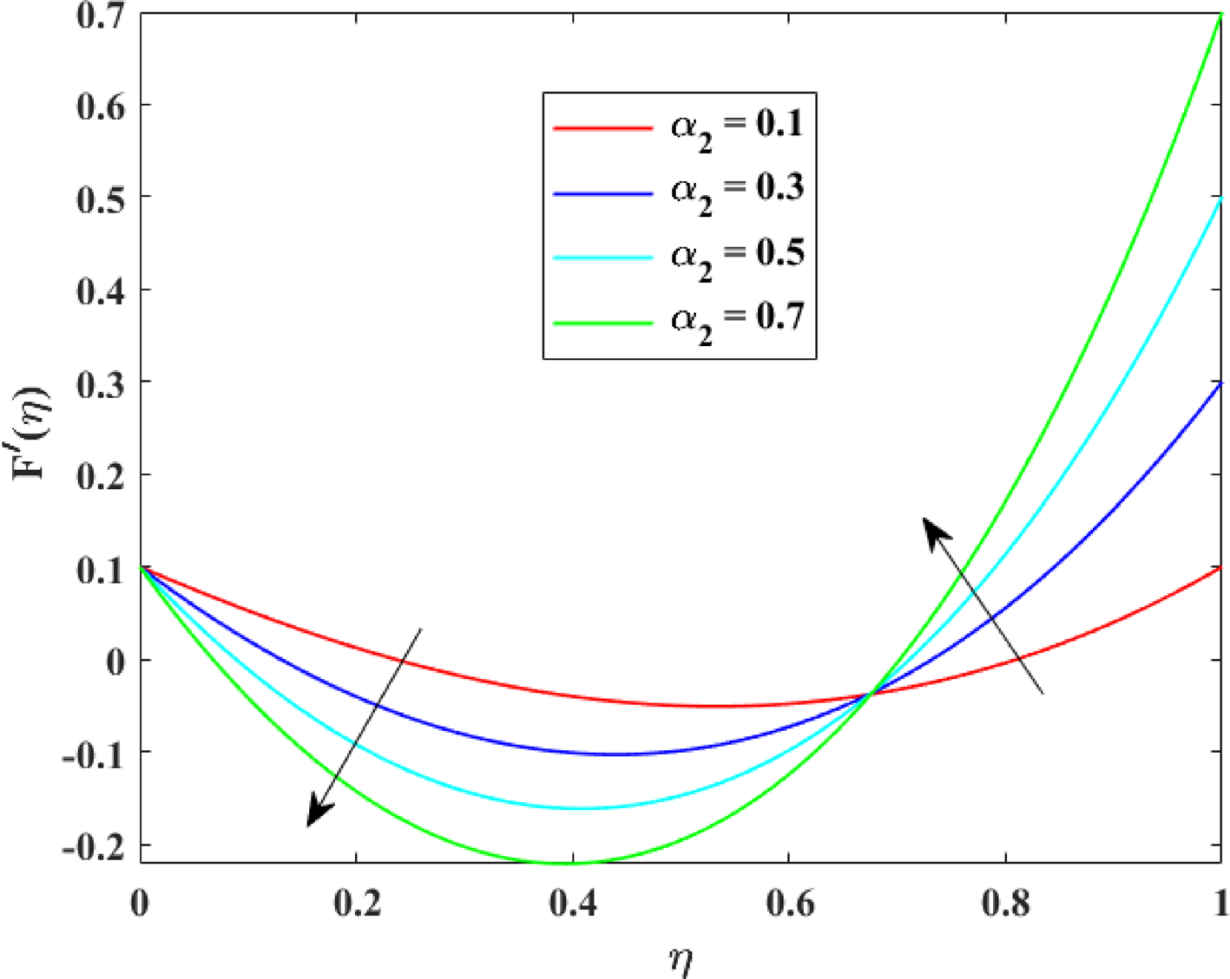
Impact of $\alpha_{2}$ on $F^{\prime}\left( \eta \right)$

**Fig. 6:**
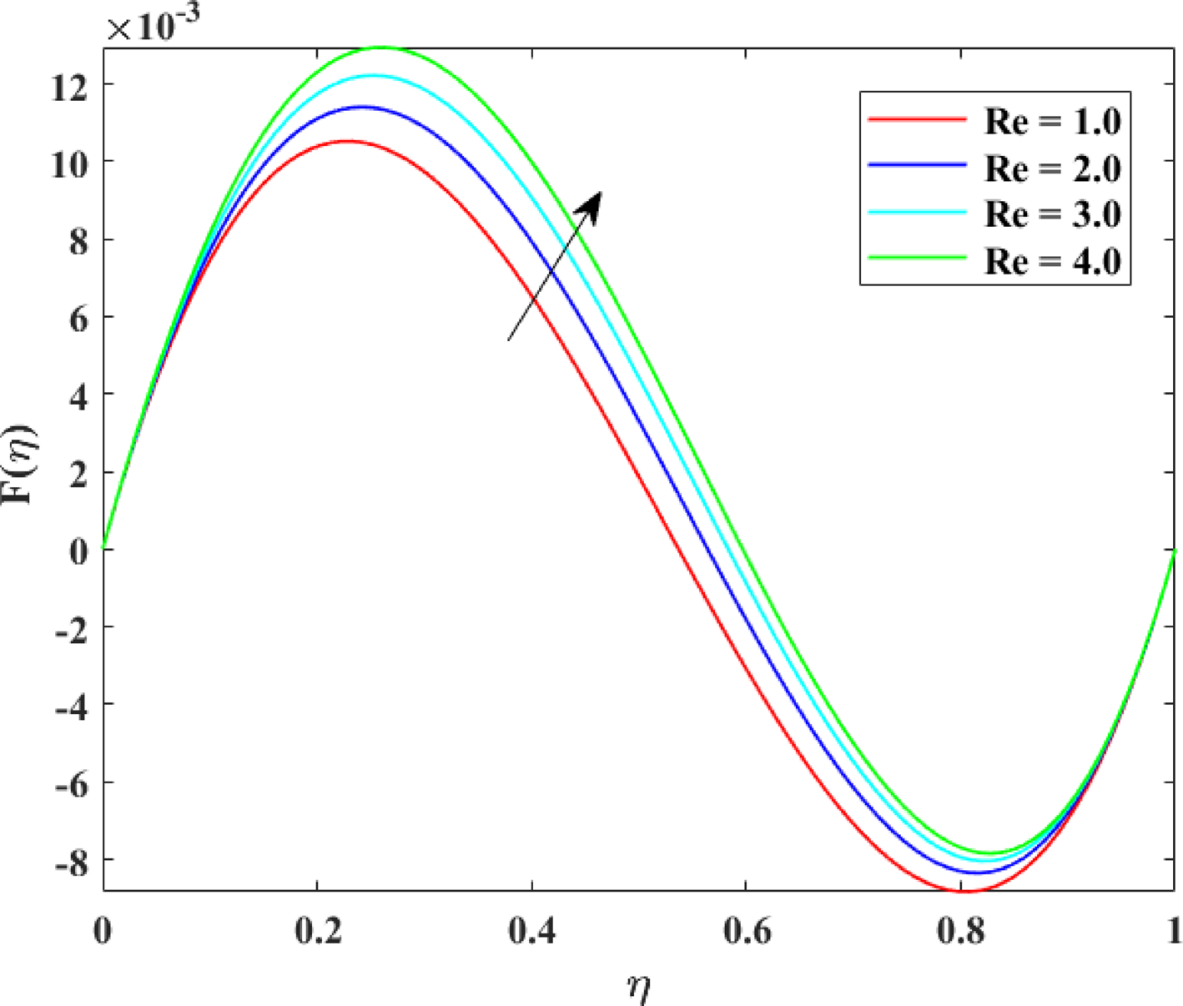
Impact of ${\mathrm{Re}}$ on $F\left( \eta \right)$

**Fig. 7:**
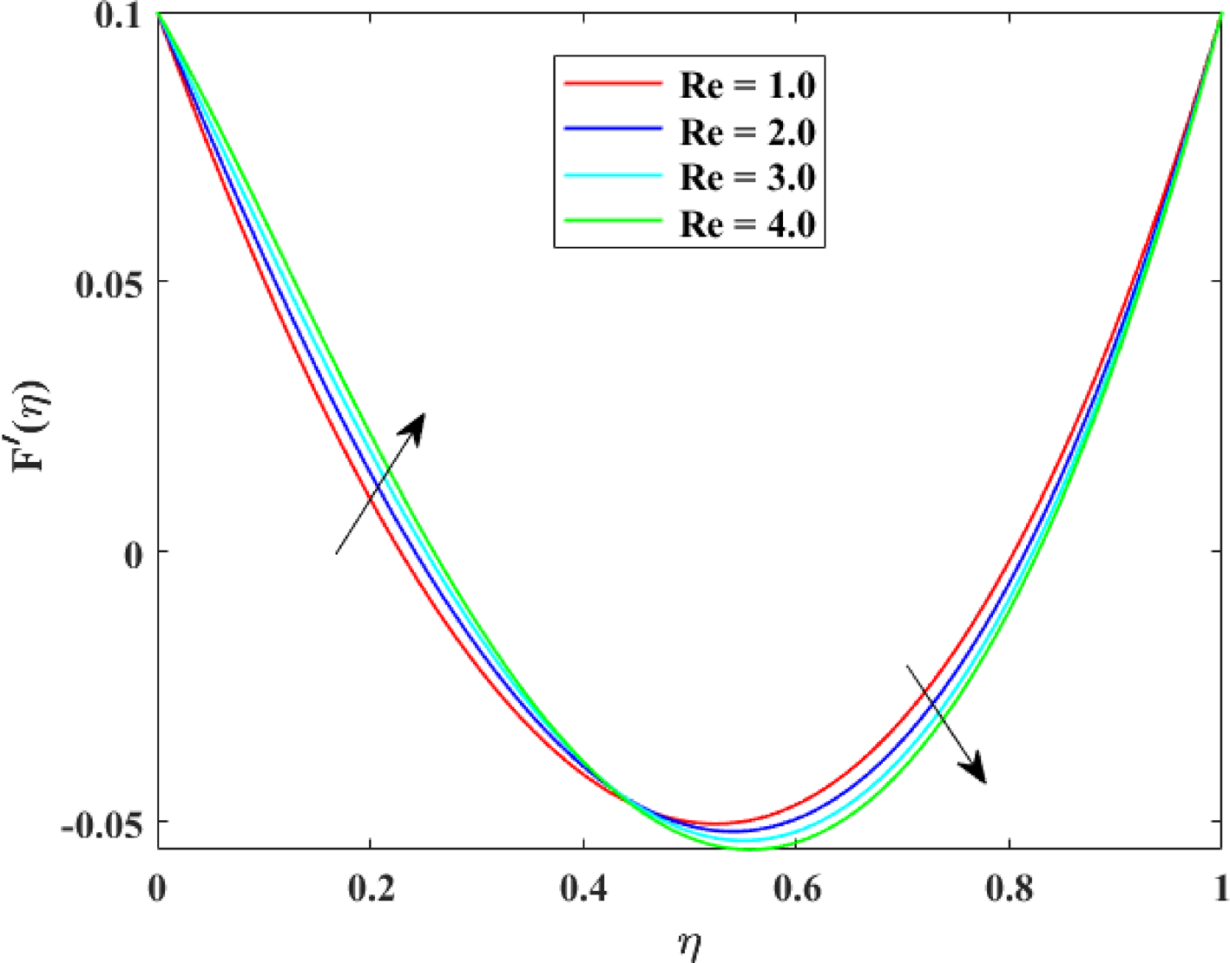
Impact of ${\mathrm{Re}}$ on $F^{\prime}\left( \eta \right)$

**Fig. 8:**
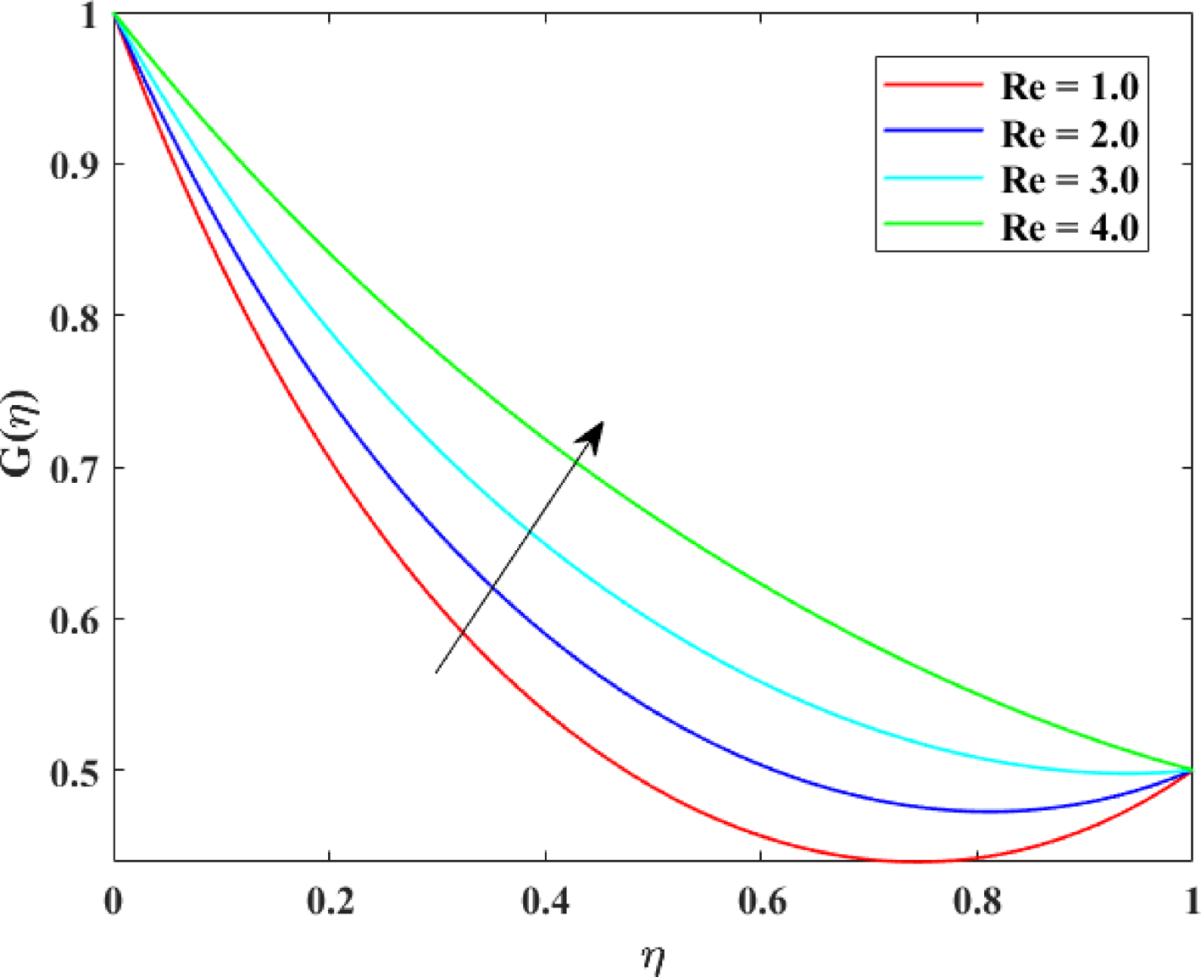
Impact of ${\mathrm{Re}}$ on $G\left( \eta \right)$

**Fig. 9:**
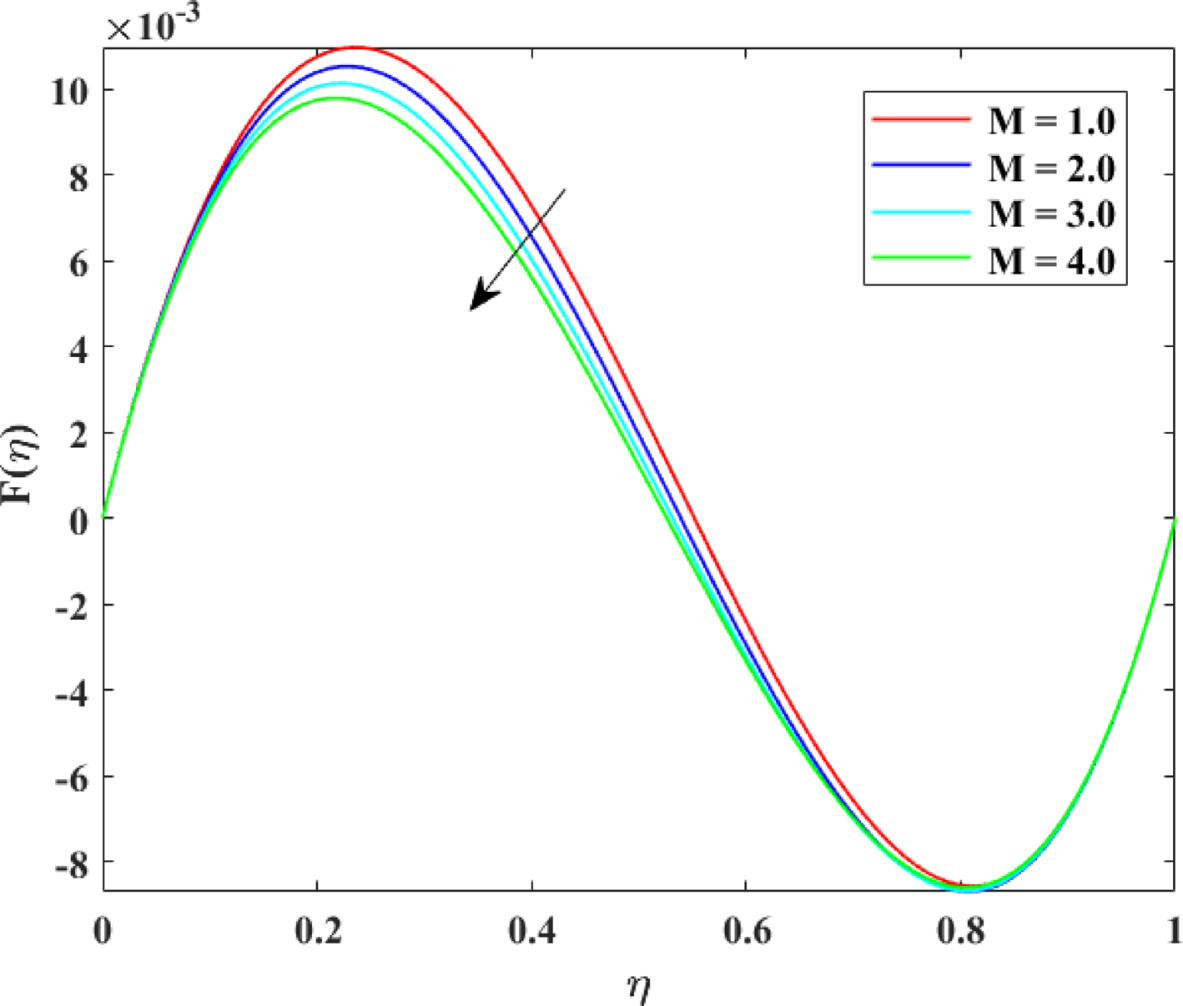
Impact of $M$ on $F\left( \eta \right)$

**Fig. 10:**
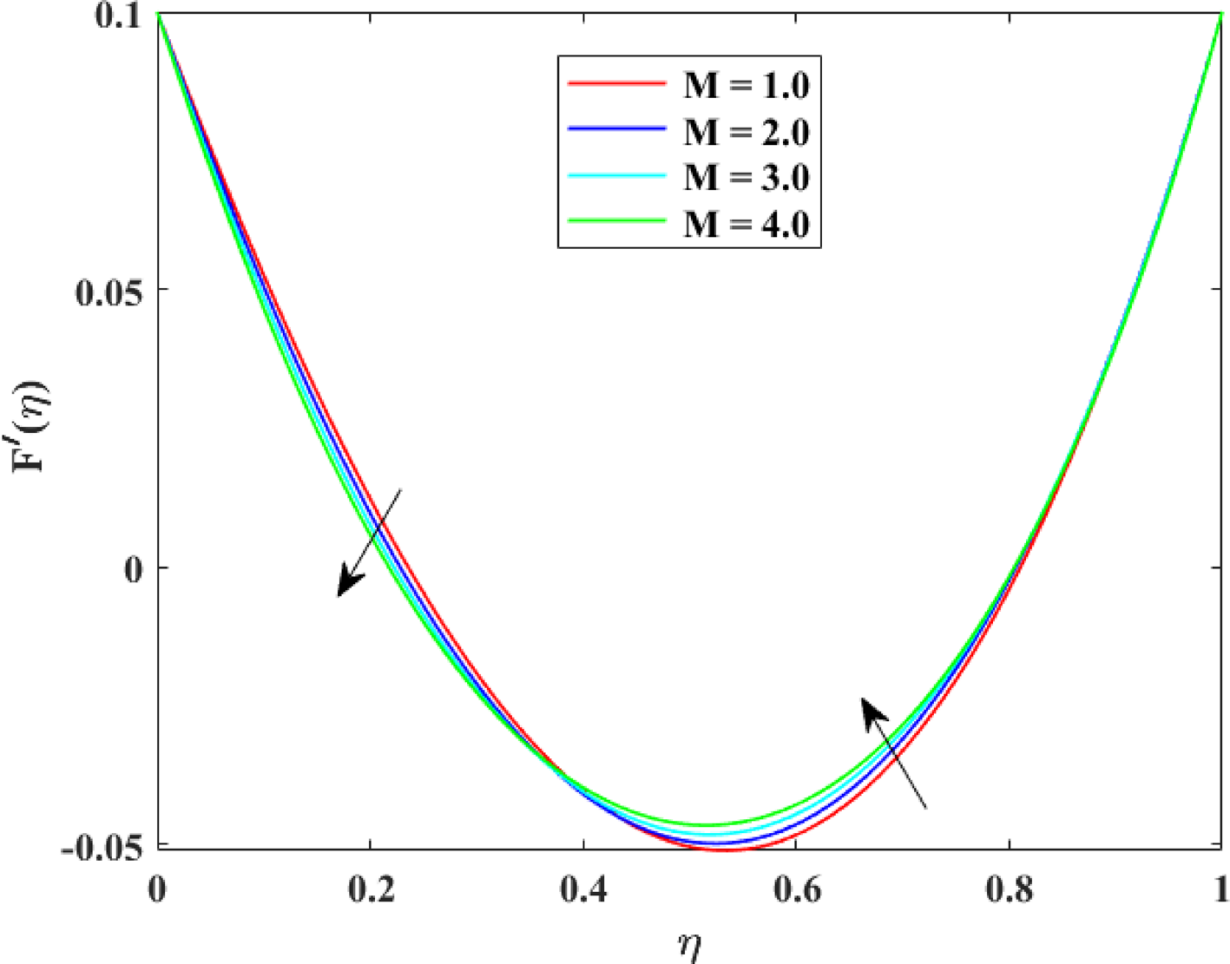
Impact of $M$ on $F^{\prime}\left( \eta \right)$

**Fig. 11:**
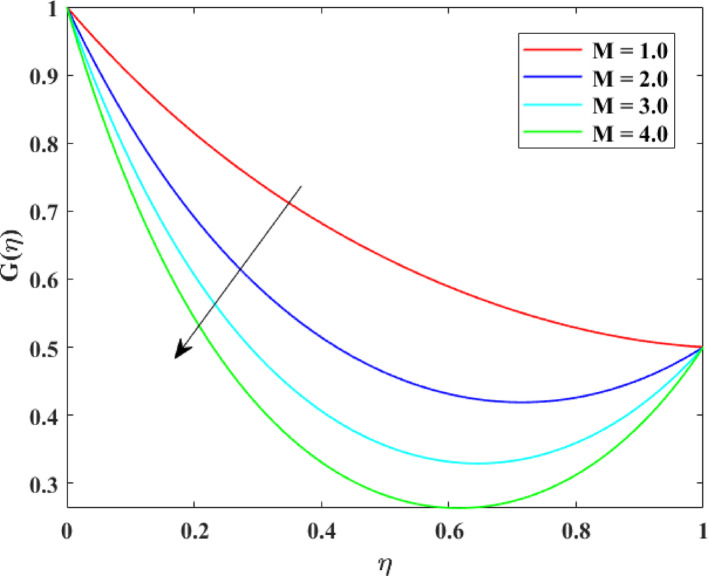
Impact of $M$ on $G\left( \eta \right)$

**Fig. 12:**
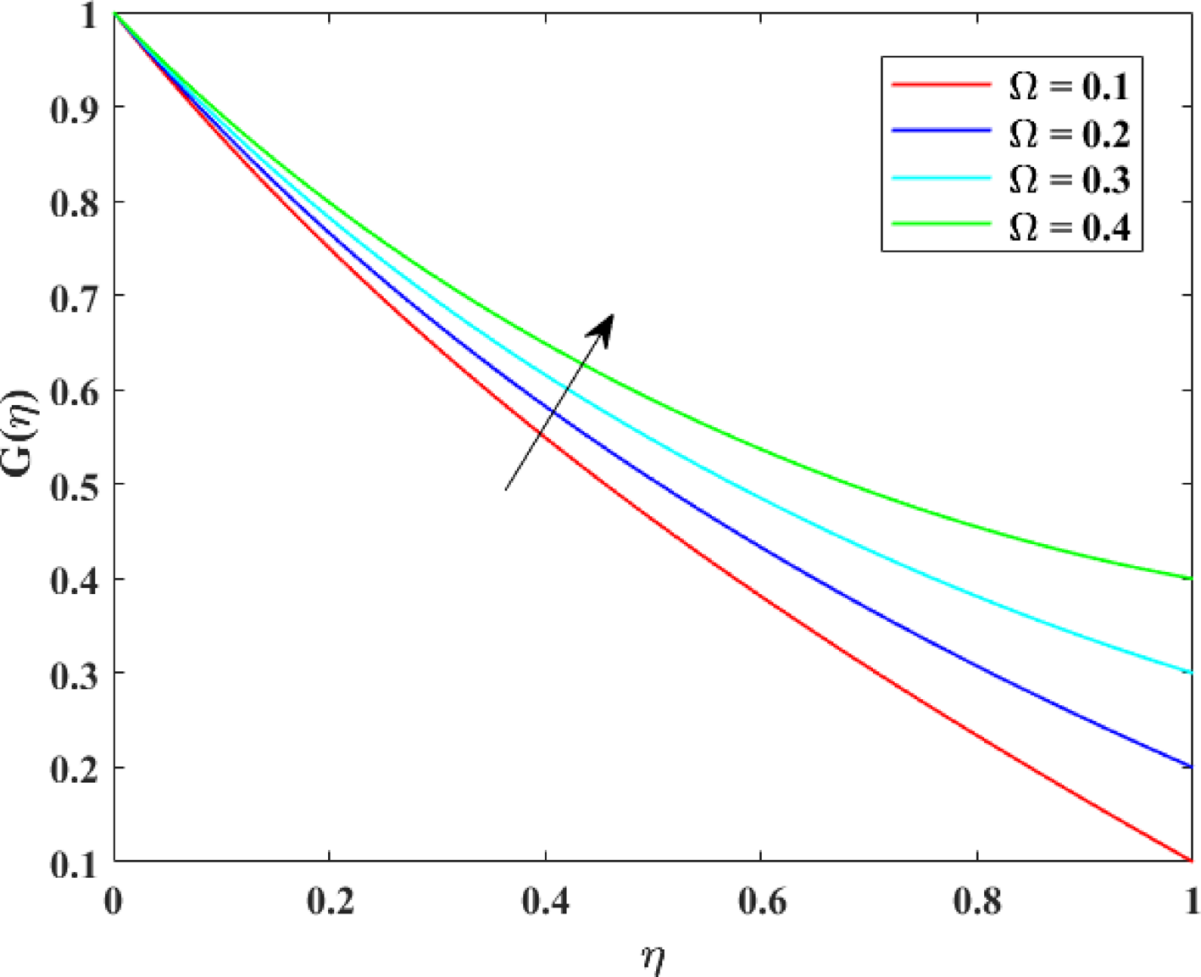
Impact of $\Omega$ on $G\left( \eta \right)$

The impression of various factors on axial velocity $\left\{ {F\left( \eta \right)} \right\}$, radial velocity $\left\{ {F^{\prime}\left( \eta \right)} \right\}$ and tangential velocity $\left\{ {G\left( \eta \right)} \right\}$ is explained in Figs. 2, 3, 4, 5, 6, 7, 8, 9, 10, 11, 12. Figure 2 illustrates the impression of stretching factor $\left( {\alpha_{1} } \right)$ at lower disk on $\left\{ {F\left( \eta \right)} \right\}$ with augmenting behavior in $\left\{ {F\left( \eta \right)} \right\}$ for surge in $\left( {\alpha_{1} } \right)$. This augmentation in $\left\{ {F\left( \eta \right)} \right\}$ with growth $\left( {\alpha_{1} } \right)$ can be physically understood as consequence of intensifies pulling action exerted by the stretching surface on the fluid. As the stretching factor increases, the surface of lower disk stretches more rapidly, imparting greater momentum to the adjacent fluid layers. This increased momentum transfer accelerates the fluid motion in the axial direction, causing a rise in $\left\{ {F\left( \eta \right)} \right\}$. Additionally, higher stretching reduces fluid resistance nearby the surface by thinning the momentum boundary layer, permitting greater fluid mobility along the axial direction. Consequently, the intensified stretching promotes faster fluid transport and strengthens the overall flow field. Figure 3 illustrates the impression of $\left( {\alpha_{1} } \right)$ on $\left\{ {F^{\prime}\left( \eta \right)} \right\}$ with dual behavior in $\left\{ {F^{\prime}\left( \eta \right)} \right\}$ for surge in $\left( {\alpha_{2} } \right)$. The twofold behavior of $\left\{ {F^{\prime}\left( \eta \right)} \right\}$ with an increase in $\left( {\alpha_{1} } \right)$ can be physically explained by the competing effects of surface stretching and flow redistribution across the gap between the disks. On the interval $0 \le \eta \le 0.3$, stronger stretching of the lower disk enhances the outward pulling force on the fluid near the disk, increasing radial momentum and causing the profiles $\left\{ {F^{\prime}\left( \eta \right)} \right\}$ to rise. However, on the interval $0.3 < \eta \le 1.0$, the intensified stretching induces greater axial transport and pressure adjustment, which redirects fluid motion away from the radial direction. This redistribution weakens the radial flow, casing a decline in $\left\{ {F^{\prime}\left( \eta \right)} \right\}$. As a result, the combined influence of stretching-induced acceleration and flow realignment produces an initial increase followed by a reduction in $\left\{ {F^{\prime}\left( \eta \right)} \right\}$. Figure 4 demonstrates the impression of stretching factor $\left( {\alpha_{2} } \right)$ on $\left\{ {F\left( \eta \right)} \right\}$ with growth in $\left\{ {F\left( \eta \right)} \right\}$ for surge in $\left( {\alpha_{2} } \right)$. The reduction in $\left\{ {F\left( \eta \right)} \right\}$ with the augmentation in $\left( {\alpha_{2} } \right)$ can be physically attributed to the redistribution of fluid momentum caused by stronger surface stretching. As the upper disk stretches more intensely, it promotes enhanced radial motion of the fluid along the disk surface. This increased radial transport diverts momentum away from the axial direction and alters the pressure distribution between the disks. Consequently, the resistance to axial motion increases, weakening the axial flow and leading to a noticeable reduction in axial velocity throughout the flow domain. Figure 5 explains the impression of stretching factor $\left( {\alpha_{2} } \right)$ at upper disk on $\left\{ {F^{\prime}\left( \eta \right)} \right\}$ with dual growth in $\left\{ {F^{\prime}\left( \eta \right)} \right\}$ for surge in $\left( {\alpha_{2} } \right)$. The observed surge in $\left\{ {F^{\prime}\left( \eta \right)} \right\}$ with increasing $\left( {\alpha_{2} } \right)$ arises from the evolving balance between near-surface resistance and momentum redistribution across the flow domain. On the $0 \le \eta \le 0.65$ interval, stronger stretching increases viscous resistance and axial–radial momentum coupling near the disk, which suppresses radial motion and causes a temporary reduction in radial velocity. However, on the interval $0.65 < \eta \le 1.0$, the intensified stretching injects greater tangential and axial momentum into the fluid, leading to enhanced outward radial transport away from the disk. This momentum redistribution overcomes viscous damping, causing rise $\left\{ {F^{\prime}\left( \eta \right)} \right\}$ in the latter region. The effects of Reynolds number $\left( {\mathrm{Re}} \right)$ on $\left\{ {F\left( \eta \right)} \right\}$, $\left\{ {F^{\prime}\left( \eta \right)} \right\}$ and $\left\{ {G\left( \eta \right)} \right\}$ are explained in Figs. 6, 7, 8. From Fig. 6, a growth in $\left\{ {F\left( \eta \right)} \right\}$ is observed, while from Fig. 7 with a dual behavior in $\left\{ {F^{\prime}\left( \eta \right)} \right\}$ is noticed and from Fig. 8 a surge in $\left\{ {G\left( \eta \right)} \right\}$ is revealed with growth in $\left( {\mathrm{Re}} \right)$. The intensification in $\left\{ {F\left( \eta \right)} \right\}$ with augmentation in the Reynolds number is physically associated with the domination of force of inertia on viscous resistance, which allows the fluid to move more freely in the axial direction. At higher $\left( {\mathrm{Re}} \right)$, enhanced fluid inertia strengthens momentum transport, leading to accelerated axial flow as revealed in Fig. 6. The dual behavior of radial velocity $\left\{ {F^{\prime}\left( \eta \right)} \right\}$ arises from the spatial redistribution of inertia: on the interval $0 \le \eta < 0.45$, stronger inertial effects amplify outward radial motion, increasing radial velocity, whereas on the interval $0.45 \le \eta \le 1.0$, viscous diffusion and pressure adjustments counteract inertia, causing a reduction in $\left\{ {F^{\prime}\left( \eta \right)} \right\}$ as explained in Fig. 7. Additionally, the growth in tangential velocity $\left\{ {G\left( \eta \right)} \right\}$ is due to declined viscous damping at higher $\left( {\mathrm{Re}} \right)$, enabling rotational effects to intensify and enhance circumferential fluid motion as given in Fig. 8. The effects of magnetic factor $\left( M \right)$ on $\left\{ {F\left( \eta \right)} \right\}$, $\left\{ {F^{\prime}\left( \eta \right)} \right\}$ and $\left\{ {G\left( \eta \right)} \right\}$ are explained in Figs. 9, 10 and 11. From Fig. 9, a decline in $\left\{ {F\left( \eta \right)} \right\}$ is observed, while from Fig. 10 with a dual behavior in $\left\{ {F^{\prime}\left( \eta \right)} \right\}$ is noticed and from Fig. 11 a reduction in $\left\{ {G\left( \eta \right)} \right\}$ is revealed with growth in $\left( M \right)$. The reduction in $\left\{ {F\left( \eta \right)} \right\}$ with an intensification in $\left( M \right)$ is physically attributed to the Lorentz force generated by magnetic effects, which opposes the motion of the electrically conducting fluid and suppresses axial momentum. This magnetic damping effect increases flow resistance, causing a reduction of the axial flow as revealed in Fig. 9. The dual features of $\left\{ {F^{\prime}\left( \eta \right)} \right\}$ arises from the non-uniform effects of the Lorentz force across the flow domain: on the interval $0 \le \eta \le 0.35$, magnetic braking dominates and weakens radial motion, causing a decay in $\left\{ {F^{\prime}\left( \eta \right)} \right\}$, while on the interval $0.35 \le \eta \le 1.0$, momentum redistribution and pressure adjustments enhance outward radial transport, resulting in an augmentation of $\left\{ {F^{\prime}\left( \eta \right)} \right\}$ as illustrated in Fig. 10. Similarly, the tangential velocity $\left\{ {G\left( \eta \right)} \right\}$ decreases due to magnetic resistance opposing rotational motion and dissipating kinetic energy as explained in Fig. 11. The impression of rotational factor $\left( \Omega \right)$ on $\left\{ {G\left( \eta \right)} \right\}$ is explained in Fig. 12 with intensifying behavior of $\left\{ {G\left( \eta \right)} \right\}$ for surge in $\left( \Omega \right)$. The surge in $\left\{ {G\left( \eta \right)} \right\}$ with augmentation in $\left( \Omega \right)$ is physically due to the stronger rotational motion imparted by the disks to the fluid. As $\left( \Omega \right)$ rises, the fluid near the disk surface experiences greater circumferential acceleration, which enhances momentum in the tangential direction. This increased rotational energy is transferred through viscous forces to adjacent fluid layers, intensifying overall tangential motion. Consequently, higher rotation strengthens the swirling flow, amplifies centrifugal effects, and rises $\left\{ {G\left( \eta \right)} \right\}$ throughout the fluid domain, reflecting the direct influence of disk rotation on circumferential fluid dynamics.

### Thermal profiles

**Fig. 13:**
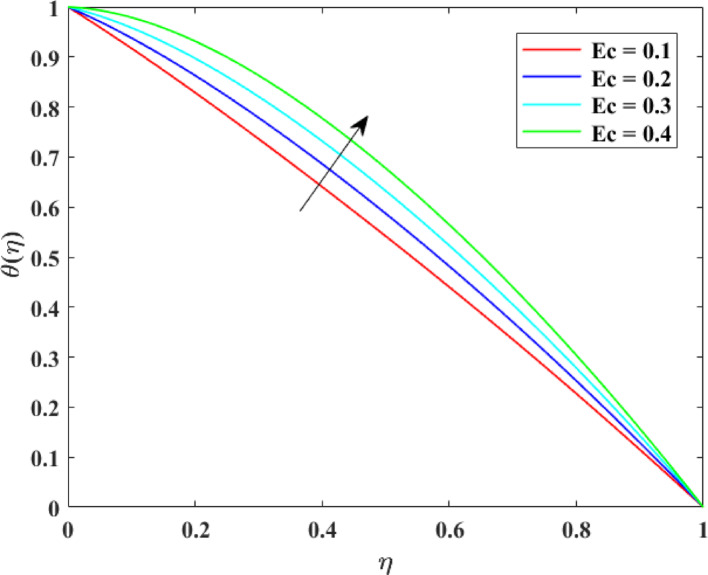
Impact of $Ec$ on $\theta \left( \eta \right)$

**Fig. 14:**
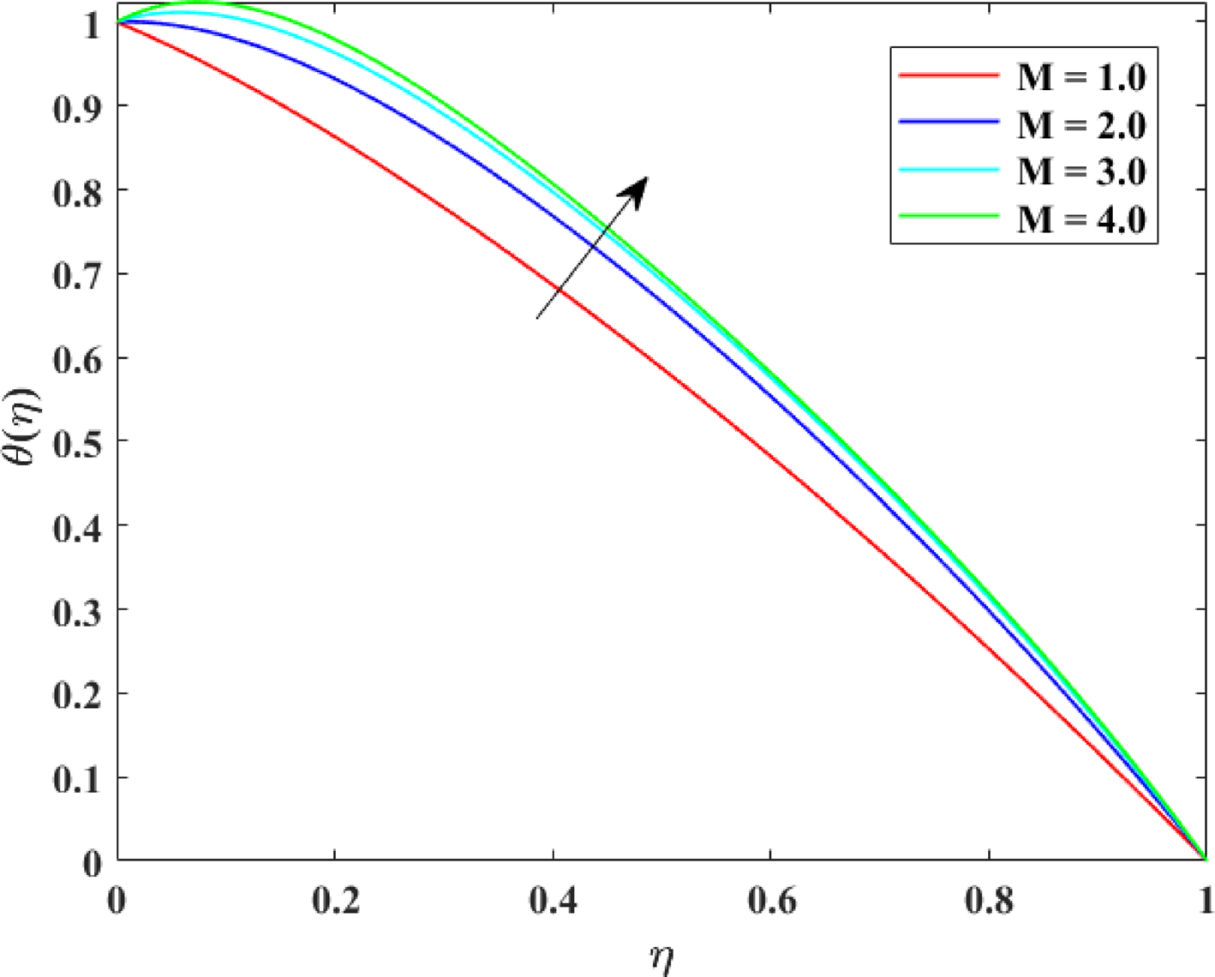
Impact of $M$ on $\theta \left( \eta \right)$

**Fig. 15:**
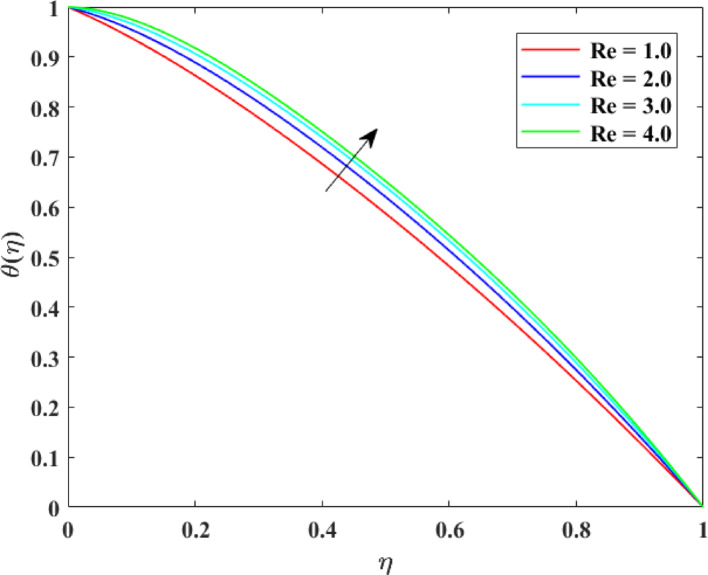
Impact of ${\mathrm{Re}}$ on $\theta \left( \eta \right)$

**Fig. 16:**
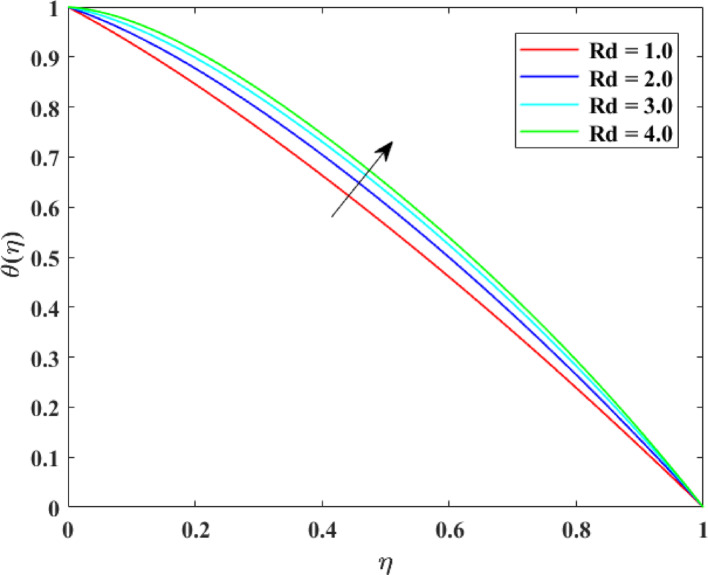
Impact of $Rd$ on $\theta \left( \eta \right)$

The impression of numerous factors on thermal profiles $\left\{ {\theta \left( \eta \right)} \right\}$ is explained in Figs. 13, 14, 15 and 16. The effect of $\left( {Ec} \right)$ on $\left\{ {\theta \left( \eta \right)} \right\}$ is explained in Fig. 13 with augmentation in $\left\{ {\theta \left( \eta \right)} \right\}$ with surge in $\left( {Ec} \right)$. The intensification in $\left\{ {\theta \left( \eta \right)} \right\}$ with augmentation in $\left( {Ec} \right)$ can be physically explained as a consequences of augmented viscous dissipation within the fluid. A higher $\left( {Ec} \right)$ indicates that kinetic energy of the fluid is being converted more effectively into inner energy due to viscous effects. This transformation generates extra heat, raising the fluid temperature. Consequently, the fluid absorbs more energy, causing an augmentation in temperature distribution. This impression is principally substantial in high-speed or high-viscosity flows, where viscous heating substantially influences thermal transport and energy characteristics. The effect of $\left( M \right)$ on $\left\{ {\theta \left( \eta \right)} \right\}$ is described in Fig. 14 with augmentation in $\left\{ {\theta \left( \eta \right)} \right\}$ for rise in $\left( M \right)$. The surge in $\left\{ {\theta \left( \eta \right)} \right\}$ with augmentation in $\left( M \right)$ can be explained physically. As $\left( M \right)$ augments, the induced electric currents within the electrically conducting fluid interact with the magnetic field, generating additional heat. This extra thermal energy advances the fluid temperature. Additionally, the Lorentz force slows down the motion of fluid, dropping convective heat transport and allowing more heat to gather in the fluid. Consequently, stronger magnetic fields enhance temperature distribution, causing an augmentation in $\left\{ {\theta \left( \eta \right)} \right\}$. The effect of $\left( {\mathrm{Re}} \right)$ on $\left\{ {\theta \left( \eta \right)} \right\}$ is illustrated in Fig. 15 with augmentation in $\left\{ {\theta \left( \eta \right)} \right\}$ for surge in $\left( {\mathrm{Re}} \right)$. The intensification in $\left( {\mathrm{Re}} \right)$ with augmentation in $\left( {\mathrm{Re}} \right)$ can be physically inferred as consequence of enhanced convective heat transport due to stronger inertial forces. Higher Reynolds numbers indicate that the fluid’s momentum dominates over viscous resistance, which accelerates the fluid motion and increases mixing within the flow. This enhanced mixing promotes more efficient transfer of thermal energy from heated surfaces into the fluid, raising the temperature distribution. Thus, higher $\left( {\mathrm{Re}} \right)$ strengthen convection, causing a rise in $\left\{ {\theta \left( \eta \right)} \right\}$. The effect of Radiation factor $\left( {Rd} \right)$ on $\left\{ {\theta \left( \eta \right)} \right\}$ is explained in Fig. 16 with augmentation in $\left\{ {\theta \left( \eta \right)} \right\}$ for surge in $\left( {Rd} \right)$. Clearly, a higher $\left( {Rd} \right)$ designates that radiative heat flux gives more significantly to the overall energy transport within the fluid. Consequently, fluid layers absorb more heat, and the temperature distribution throughout the domain rises. Consequently, stronger radiation leads to an overall augmentation in thermal profiles $\left\{ {\theta \left( \eta \right)} \right\}$.

### Concentration profiles

**Fig. 17:**
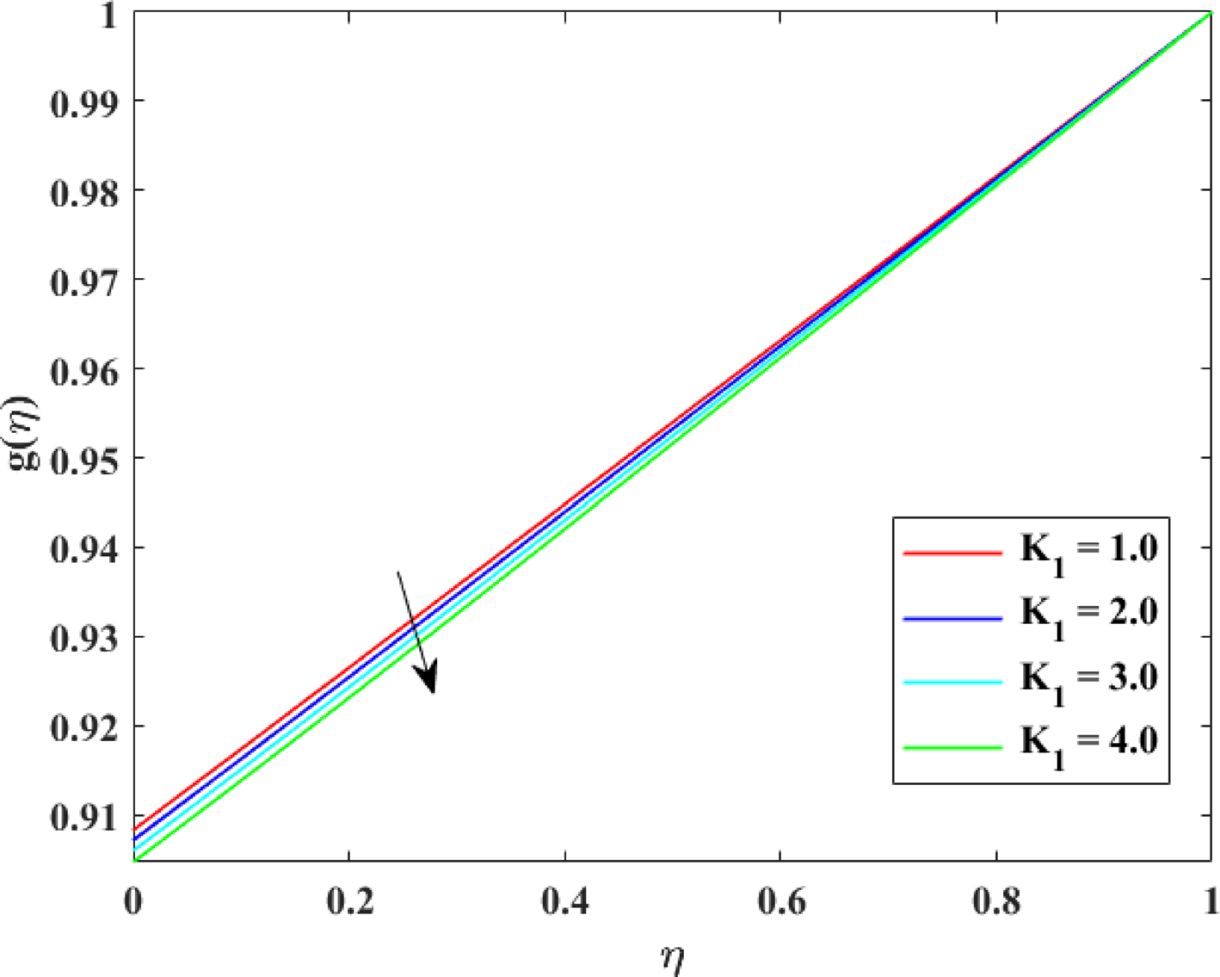
Impact of $K_{1}$ on $g\left( \eta \right)$

**Fig. 18:**
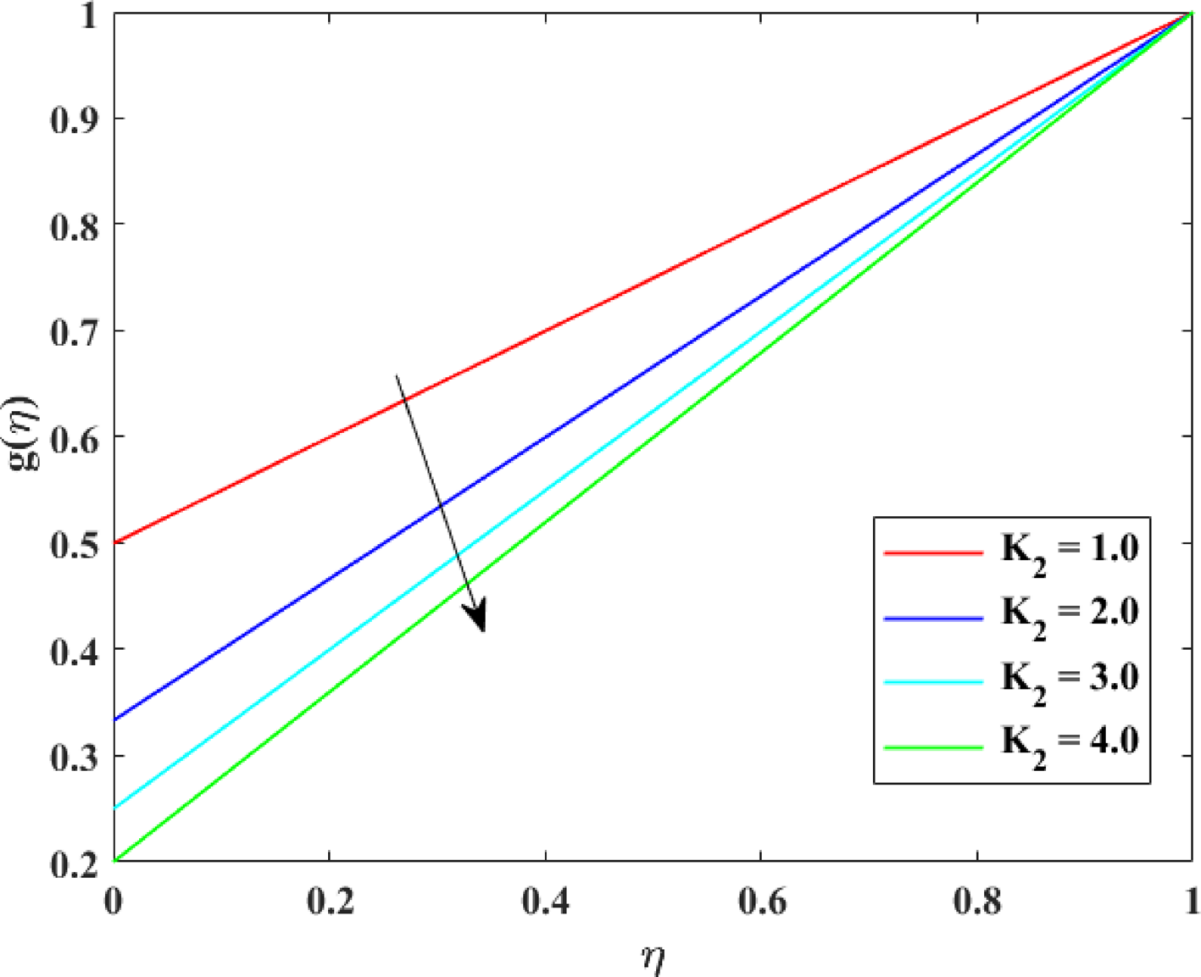
Impact of $K_{2}$ on $g\left( \eta \right)$

**Fig. 19:**
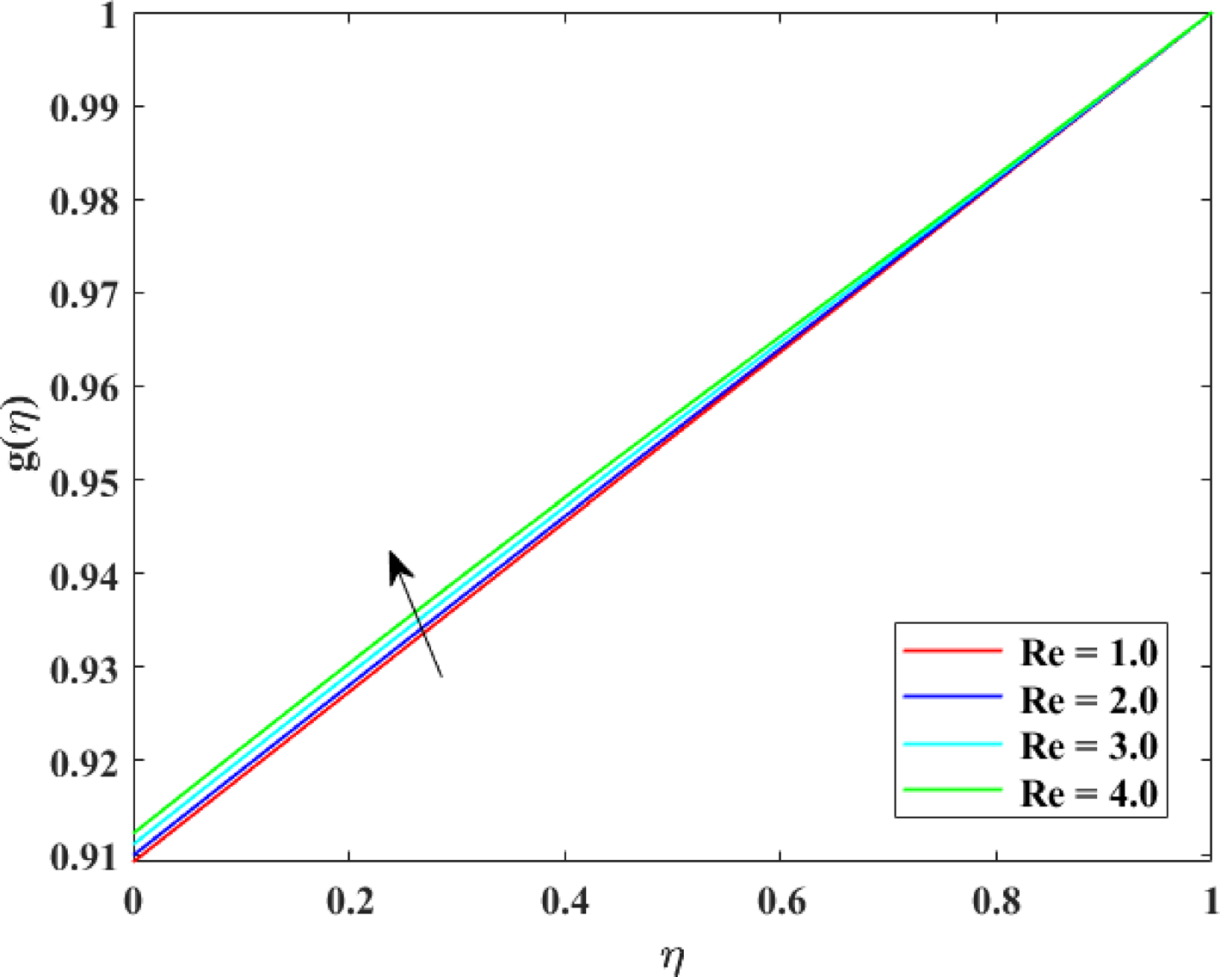
Impact of ${\mathrm{Re}}$ on $g\left( \eta \right)$

**Fig. 20:**
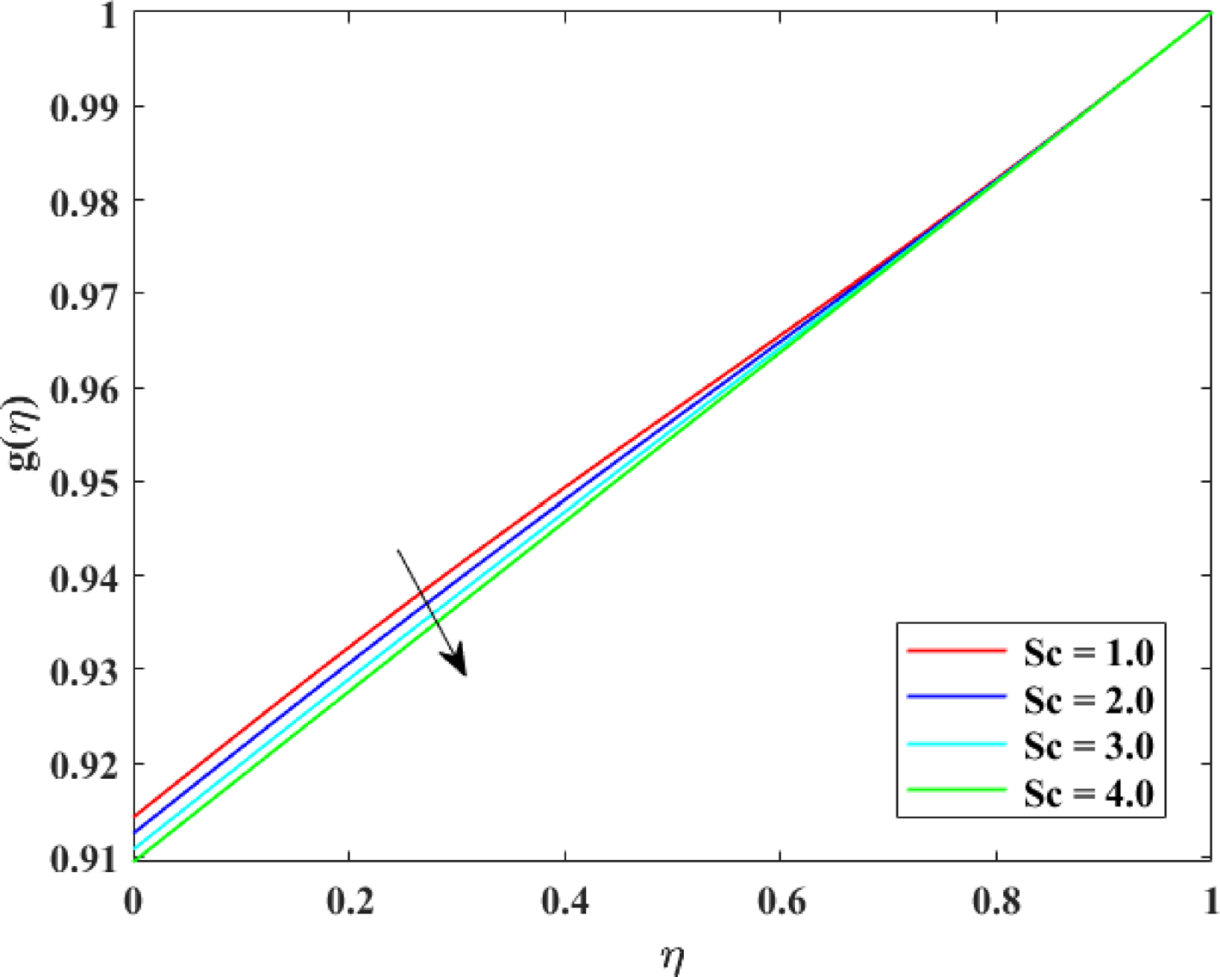
Impact of $Sc$ on $g\left( \eta \right)$

The impression of various factors concentration profiles $\left\{ {g\left( \eta \right)} \right\}$ is explained in Figs. 17, 18, 19 and 20. The effect of homogenous factor $\left( {K_{1} } \right)$ on $\left\{ {g\left( \eta \right)} \right\}$ is explained in Fig. 17 with decline in $\left\{ {g\left( \eta \right)} \right\}$ for surge in $\left( {K_{1} } \right)$. The reduction in $\left\{ {g\left( \eta \right)} \right\}$ with augmentation in $\left( {K_{1} } \right)$ is physically attributed to the intensified chemical reaction occurring within the fluid bulk. As $\left( {K_{1} } \right)$ intensifies, reactant species are consumed more rapidly throughout the flow domain, leading to a decrease in their concentration levels. This enhanced reaction activity accelerates mass transfer from the fluid to reaction products, thinning the concentration layer at boundary. Consequently, the availability of reactant species diminishes, resulting in an overall reduction in concentration profiles across the fluid region. The effect of heterogeneous factor $\left( {K_{2} } \right)$ on $\left\{ {g\left( \eta \right)} \right\}$ is explained in Fig. 18 with decline in $\left\{ {g\left( \eta \right)} \right\}$ for surge in $\left( {K_{2} } \right)$. The reduction in $\left\{ {g\left( \eta \right)} \right\}$ with augmentation in $\left( {K_{2} } \right)$ can be physically explained by the enhanced surface reaction rate at the solid–fluid interface. As $\left( {K_{2} } \right)$ surges, reactant species are consumed more rapidly at the boundary, reducing their concentration near the surface. This intensified surface consumption creates a stronger concentration gradient, which drives mass diffusion toward the surface and further depletes species from the fluid. Therefore, the concentration layer at borderline becomes thinner, and the overall concentration levels decrease throughout the flow domain due to increased reactant utilization at the interface. The effect of $\left( {\mathrm{Re}} \right)$ on $\left\{ {g\left( \eta \right)} \right\}$ is described in Fig. 19 with augmentation in $\left\{ {g\left( \eta \right)} \right\}$ for surge in $\left( {\mathrm{Re}} \right)$. The augmentation in $\left\{ {g\left( \eta \right)} \right\}$ with growth in $\left( {\mathrm{Re}} \right)$ can be physically attributed to the dominance of force of inertia on viscous effects, which enhances fluid motion and convective mass transport. At higher Reynolds numbers, stronger advection carries species more effectively away from the reactive surface into the bulk fluid, reducing residence time near the boundary where consumption may occur. This improved mixing weakens concentration gradients and thickens the concentration layer at borderline, leading to higher concentration. Consequently, increased $\left( {\mathrm{Re}} \right)$ promotes mass transport and results in augmented $\left\{ {g\left( \eta \right)} \right\}$. The effect of $\left( {Sc} \right)$ on $\left\{ {g\left( \eta \right)} \right\}$ is explained in Fig. 20 with decline in $\left\{ {g\left( \eta \right)} \right\}$ for surge in $\left( {Sc} \right)$. The reduction in $\left\{ {g\left( \eta \right)} \right\}$ with augmentation in $\left( {Sc} \right)$ can be physically interpreted as a consequence of decreased mass diffusivity relative to momentum diffusivity. A higher Schmidt number indicates weaker species diffusion within the fluid, which limits the transport of solute from the boundary into the bulk region. As a result, reactant species are less effectively replenished and are more rapidly depleted near reactive surfaces. This diminished diffusion leads to a thinner concentration layer at borderline and lower concentration levels throughout the flow domain, causing an overall reduction in $\left\{ {g\left( \eta \right)} \right\}$.

### Comparative analysis for mono, hybrid and trihybrid nanofluids

**Fig. 21:**
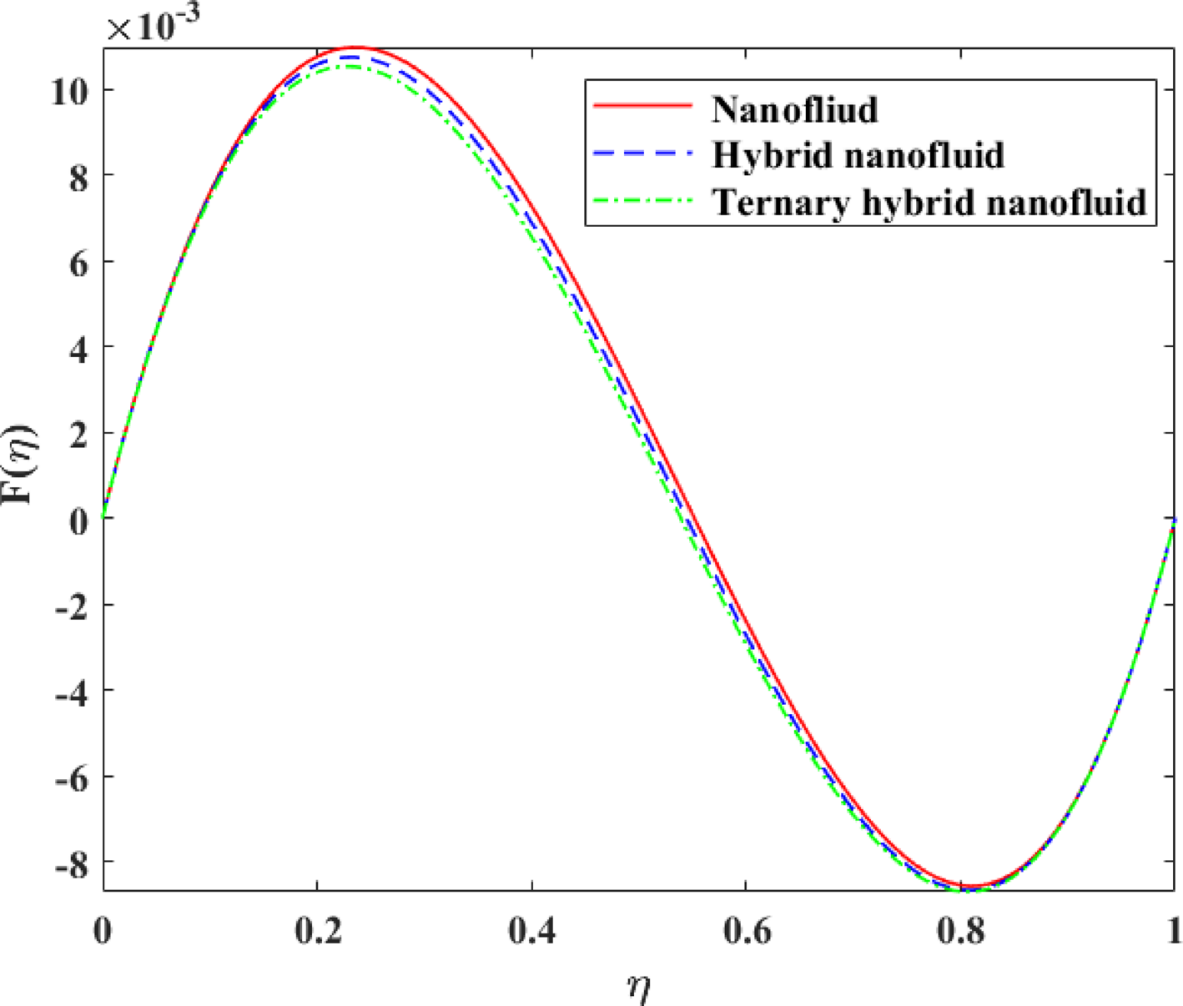
Comparison among nanofluid, hybrid nanofluid and ternary hybrid nanofluid flow for $F\left( \eta \right)$

**Fig. 22:**
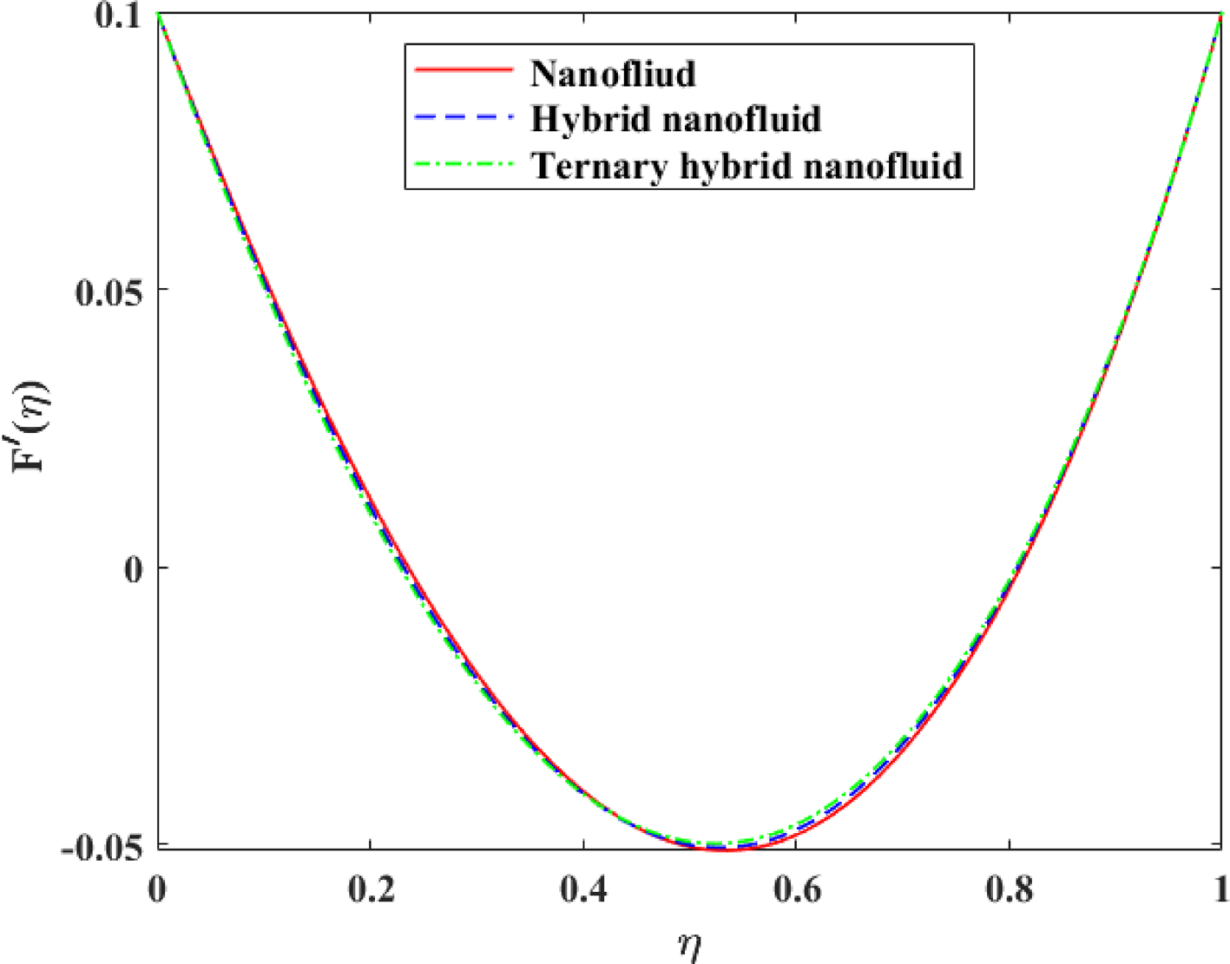
Comparison among nanofluid, hybrid nanofluid and ternary hybrid nanofluid flow for $F^{\prime}\left( \eta \right)$

**Fig. 23:**
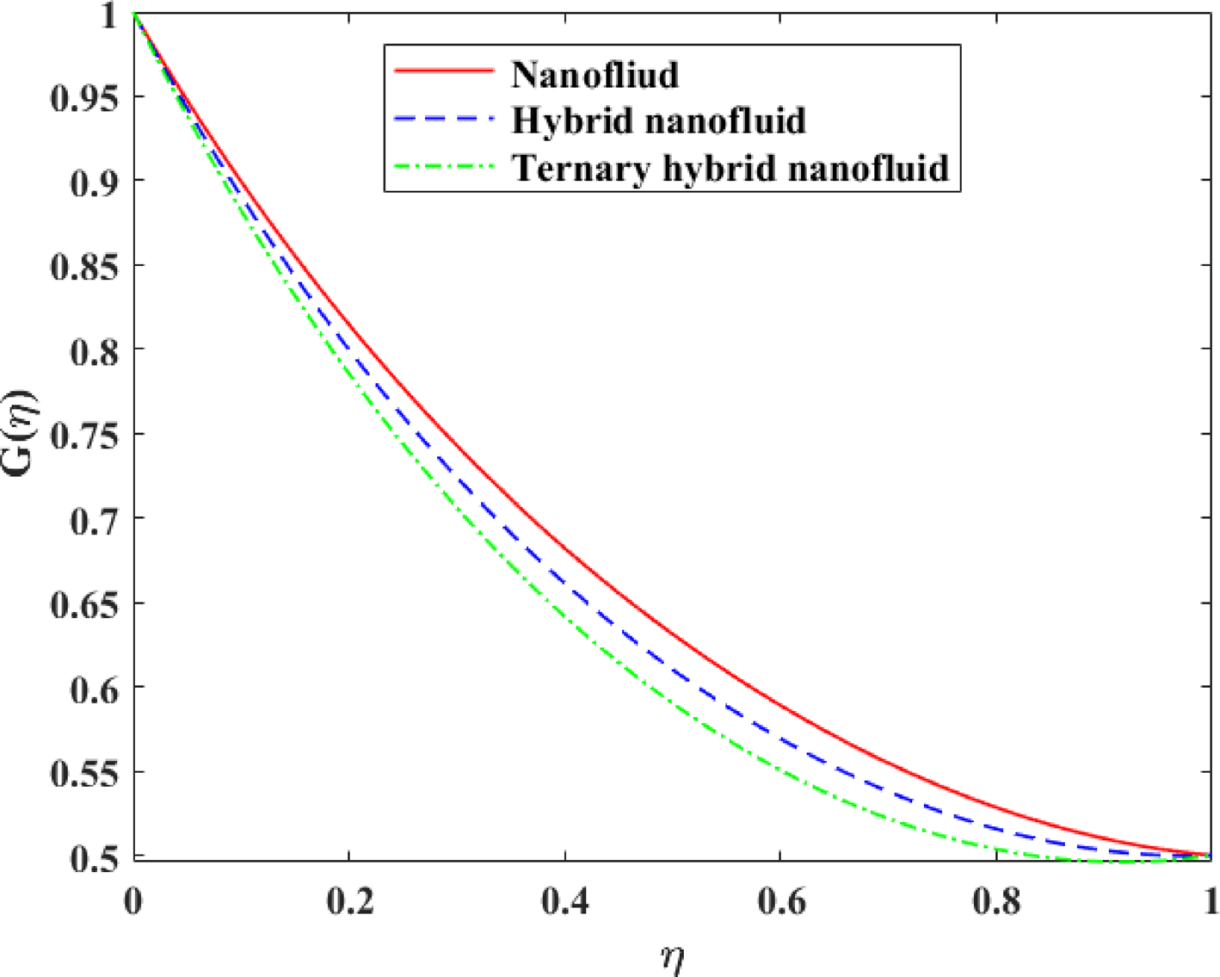
Comparison among nanofluid, hybrid nanofluid and ternary hybrid nanofluid flow for $G\left( \eta \right)$

**Fig. 24:**
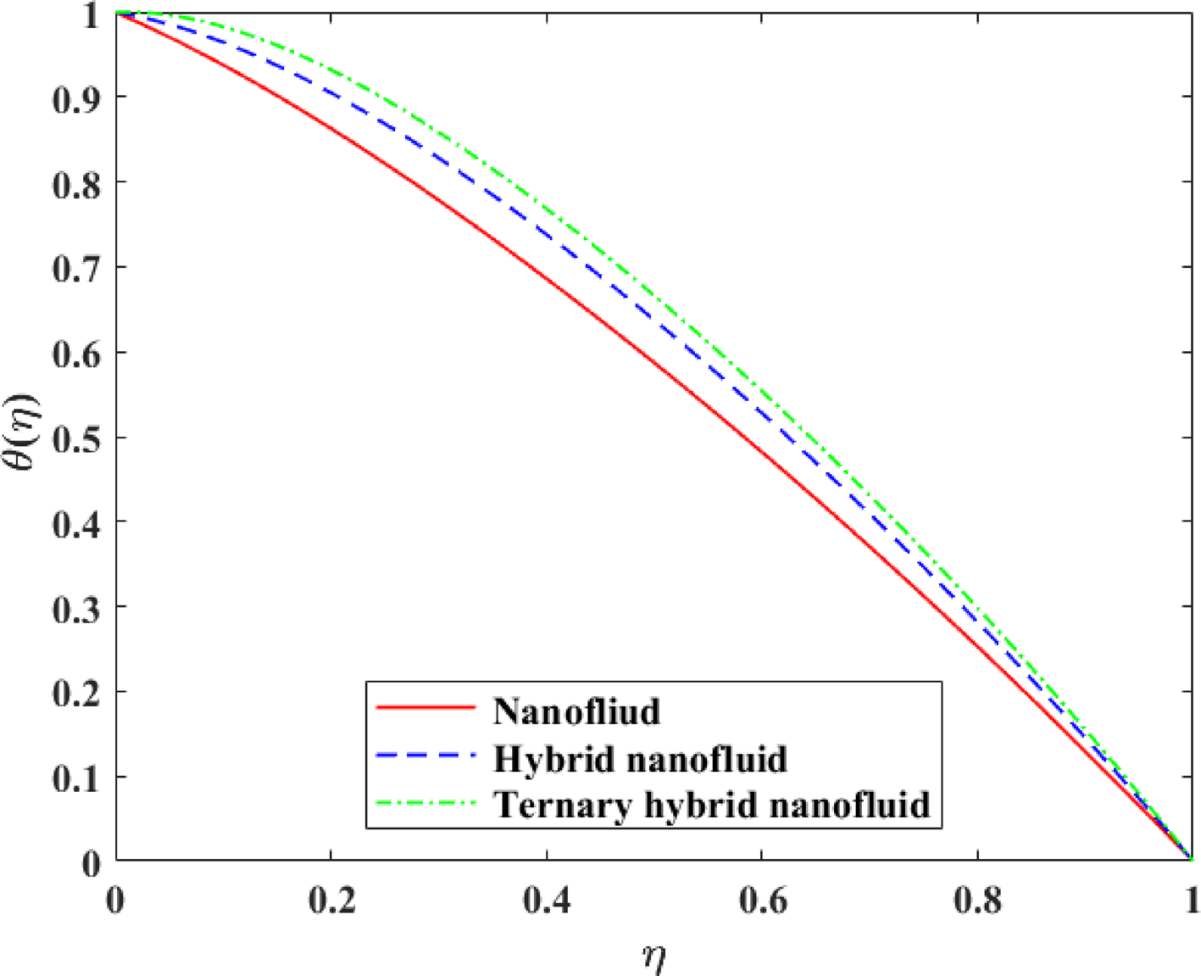
Comparison among nanofluid, hybrid nanofluid and ternary hybrid nanofluid flow for $\theta \left( \eta \right)$

**Fig. 25:**
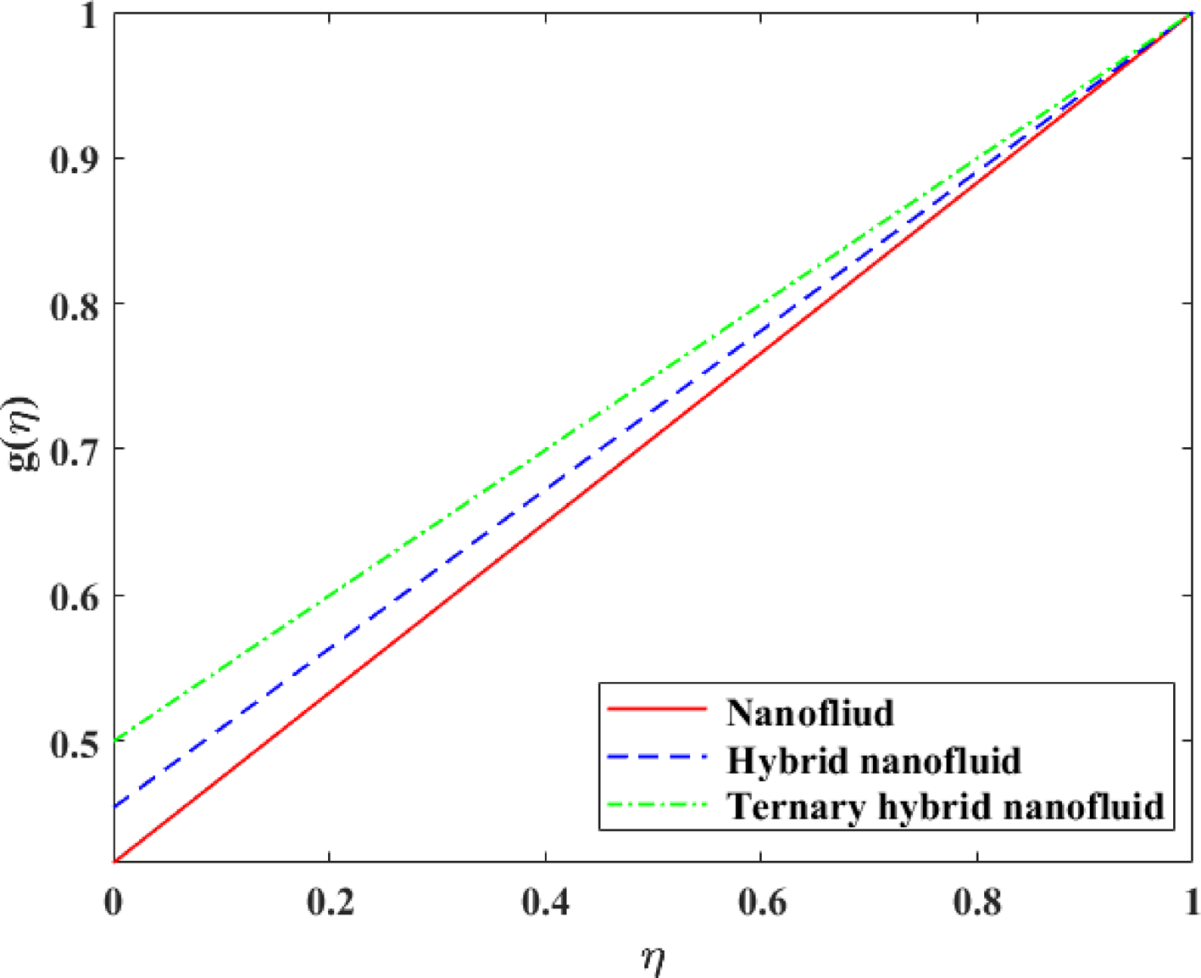
Comparison among nanofluid, hybrid nanofluid and ternary hybrid nanofluid flow for $g\left( \eta \right)$

Comparative analysis about changes in $\left\{ {F\left( \eta \right)} \right\},\,\,\left\{ {F^{\prime}\left( \eta \right)} \right\},\,\,\left\{ {G\left( \eta \right)} \right\},\,\,\left\{ {\theta \left( \eta \right)} \right\}$ and $\left\{ {g\left( \eta \right)} \right\}$ in terms of the effect of mono, hybrid and trihybrid nanofluids is discussed on Figs. 21, 22, 23, 24 and 25. Trihybrid nanofluids have a more noticeable adjustment in velocity fields than mono and hybrid nanofluids because of the progressive improvement of effective thermal conductivity and changed rheological properties, meaning that they have a greater momentum transportation capacity and flow management. The axial and tangential velocities tend to increase with the complexity of nanoparticles as they undergo better energy exchange modes whereas radial velocity experiences significant redistribution as a result of better inertia and particle interactions. The trihybrid nanofluids exhibit great enhancement in thermal profiles, which is also a result of the improved heat transfer capability due to increased thermal conductivity and enhanced diffusion of energy at the micro-scale. On the other hand, concentration profiles exhibit higher gradients in trihybrid nanofluids, which can be explained by greater mass diffusivity, and the contact between particles and fluids. Generally, the findings prove trihybrid nanofluids are superior to mono and hybrid nanofluids in controlling the flow behavior, heat transfer, and the transportation of species, and thus are very beneficial in the development of high-level thermal and fluid engineering applications.

### Stream-lines and contour-lines analysis

**Fig. 26:**
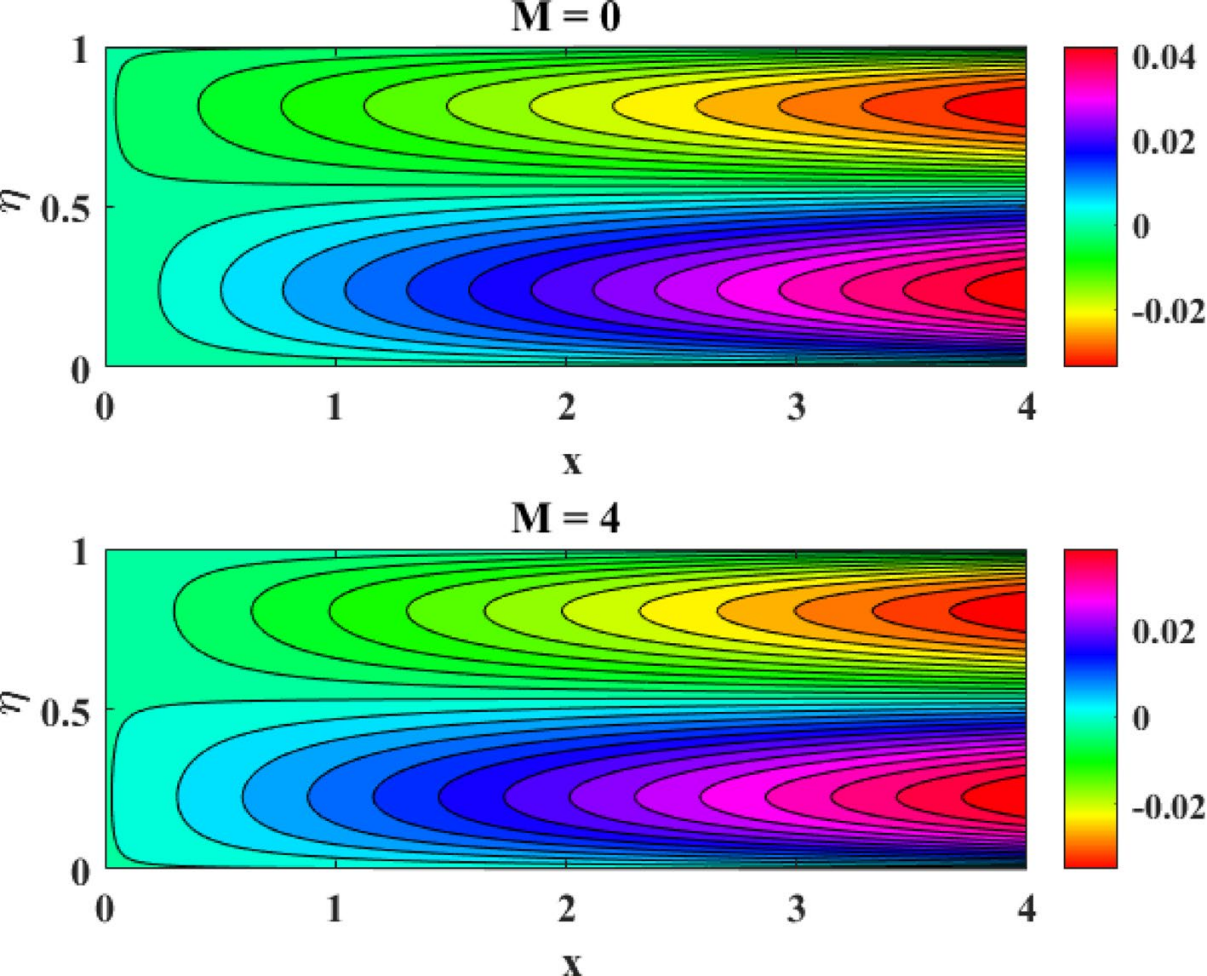
Streamline behavior of the ternary hybrid nanofluid flow

**Fig. 27:**
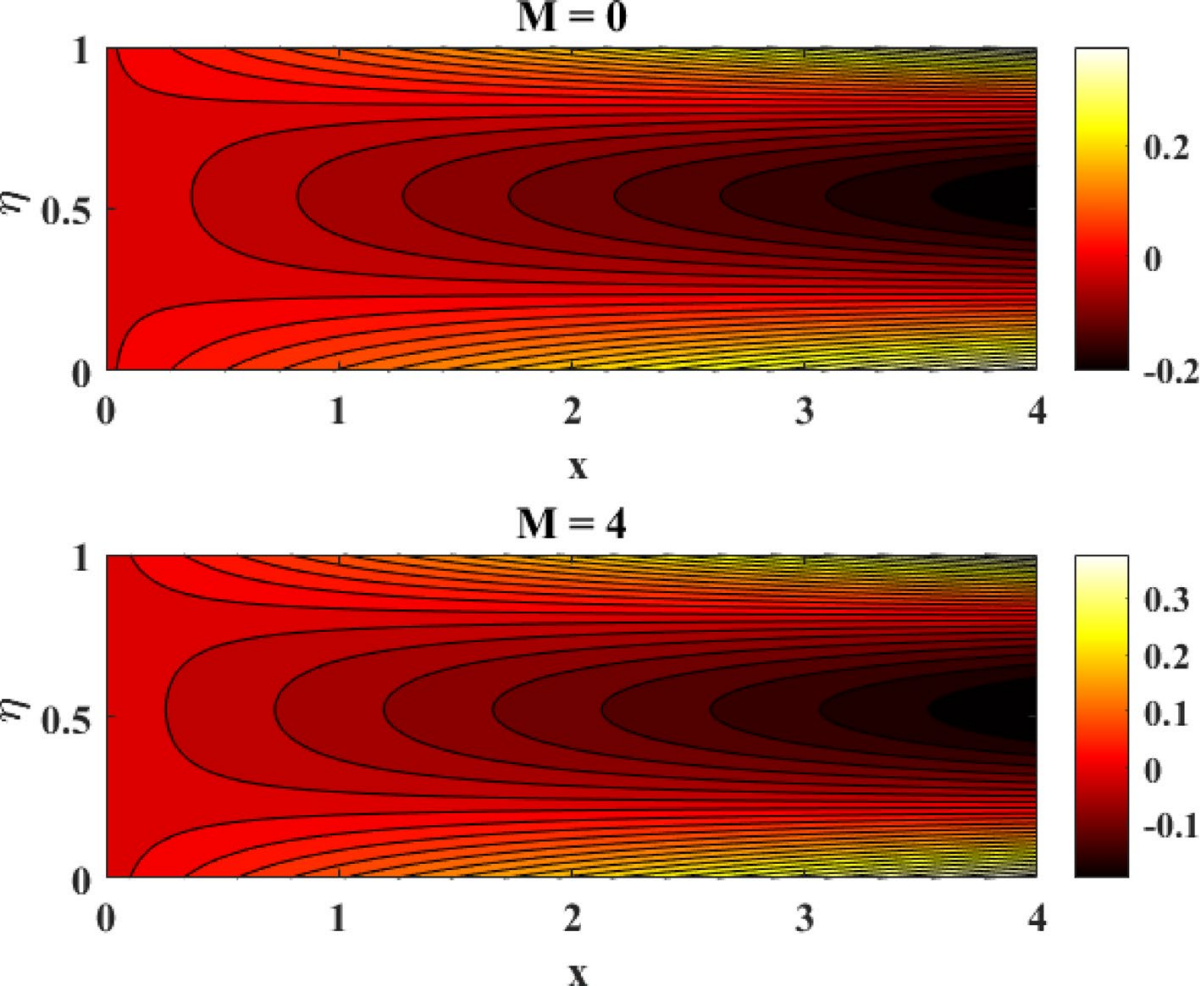
Contour lines of the ternary hybrid nanofluid flow

Figure 26 illustrates the influence of the magnetic factor on the streamline patterns of a ternary hybrid nanofluid by comparing the cases of (M = 0) and (M = 4). In the absence of a magnetic field (M = 0), the streamlines are smooth, widely spaced, and exhibit stronger circulation, indicating higher fluid mobility and dominant inertial effects. However, when the magnetic factor is increased to (M = 4), the streamlines become denser and more compressed near the disk surfaces, reflecting the suppressive role of the Lorentz force. This magnetic damping resists fluid motion, weakens circulation strength, and reduces flow intensity. The altered streamline topology confirms that a stronger magnetic field effectively controls and stabilizes the flow of ternary hybrid nanofluids, which is particularly beneficial in applications requiring regulated momentum transport and enhanced flow stability. Figure 27 demonstrates the effect of the magnetic factor on the contour-line distribution for a ternary hybrid nanofluid by contrasting the cases (M = 0) and (M = 4). When no magnetic field is applied (M = 0), the contour lines are more widely spaced, indicating higher velocity and temperature gradients and stronger convective transport within the flow domain. As the magnetic factor increases to (M = 4), the contour lines become more closely packed and uniformly distributed, reflecting the damping influence of the Lorentz force. This magnetic resistance suppresses fluid motion, reduces gradient intensity, and promotes a more stabilized flow structure. The observed contour patterns confirm that the magnetic field significantly governs energy and momentum distribution in ternary hybrid nanofluids.

### Tables discussion

Table 1 explains the numerical values of thermo-physical features. Table 2, 3 and 4 thermophysical properties of mono, hybrid and trihybrid nanofluids. Table 5 illustrates comparison of the present work with the published work.

Table 6 examines the effects of various factors on Nussselt numbers ($Nu_{1}$,$Nu_{2}$) at lower and upper disks. The observed augmentation in ($Nu_{1}$,$Nu_{2}$) with increasing magnetic factor, Eckert number, lower-disk stretching factor, and radiation factor indicates an overall enhancement in heat transfer due to stronger thermal energy generation and transport mechanisms. The magnetic field intensifies Joule heating, the Eckert number represents increased viscous dissipation, stretching of the lower disk improves convective transport, and radiation adds additional thermal energy to the system. The more pronounced increase in the Nusselt number at the upper disk can be physically attributed to the cumulative upward transport of heat and the stronger thermal gradients formed near the upper surface. This results in enhanced heat flux and more effective thermal exchange at the upper disk compared to the lower disk.

**Table 6:** Impact of $M$,$Ec$,$\alpha_{1}$, and $Rd$ on Nussselt numbers ($Nu_{1}$, $Nu_{2}$)

$M$	$Ec$	$\alpha_{1}$	$Rd$	$Nu_{1}$	$Nu_{2}$
0.3				0.53197	1.41673
0.4				0.52133	1.44365
0.5				0.51090	1.47108
	0.2			0.66189	1.26934
	0.3			0.64137	1.29790
	0.4			0.62149	1.32711
		0.2		0.42871	1.20355
		0.3		0.44710	1.20927
		0.4		0.46188	1.21721
			0.2	0.51832	1.26192
			0.3	0.52816	1.27327
			0.4	0.53820	1.28473

Table 7 examines the effects of various factors on Sherwood numbers ($Sh_{1}$,$Sh_{2}$). The intensification in ($Sh_{1}$,$Sh_{2}$) with rising Schmidt number and homogeneous-heterogeneous reaction factors reflects enhanced mass transfer due to stronger species gradients near the disk surfaces. A higher Schmidt number reduces mass diffusivity, intensifying concentration gradients and thereby increasing mass flux. Similarly, stronger homogeneous and heterogeneous reactions accelerate species consumption in the fluid and at the surface, further steepening concentration gradients. The more pronounced augmentation of the Sherwood number at the lower disk can be attributed to its closer interaction with the incoming flow and higher residence time of species near this surface. As a result, stronger concentration gradients develop at the lower disk, leading to greater mass transfer rates compared to the upper disk.

**Table 7:** Impact of $Sc$,$K_{1}$, and $K_{2}$ on Sherwood numbers ($Sh_{1}$, $Sh_{2}$)

$Sc$	$K_{1}$	$K_{2}$	$Sh_{1}$	$Sh_{2}$
0.2			1.34190	0.24613
0.3			1.38215	0.24982
0.4			1.42363	0.25357
	0.2		1.14291	0.15289
	0.3		1.15891	0.15442
	0.4		1.17513	0.15596
		0.2	1.05883	0.19306
		0.3	1.07895	0.19460
		0.5	1.09945	0.19616

## Conclusions

This study examines tri-hybrid nanofluid flow among two gyrating disks with combined effects of mixed convection and Joule heating. The physical model captures the complex interaction between rotation-induced forces, thermal transport, and reactive species within the nanofluid. The coupled leading equations in dimension-free form are solved efficiently using the MATLAB built-in solver bvp4c, which ensures high accuracy and numerical stability. The impression of various factors on velocity and temperature panels have discussed in upcoming paragraphs. After detailed examination of the work it has revealed that:

- With surge in starching factor at lower disk there is surge in axial flow while radial flow exhibits two fold behavior, on the interval $0 \le \eta \le 0.3$, it enhances, however on the interval $0.3 < \eta \le 1.0$ it reduces.
- With surge in starching factor at upper disk there is decline in axial flow while radial flow exhibits two fold behavior, on the interval $0 \le \eta \le 0.65$, it declines, however on the interval $0.65 < \eta \le 1.0$ it augments.
- Both axial and tangential flows augment with surge in Reynolds number while decline with growth in magnetic factor. However, in this phenomenon radial flow again exhibits two fold behavior.
- Tangential flow is supported by intensification in rotational factor.
- Thermal profiles augment with escalation in Eckert, Reynolds numbers, magnetic and radiation factors.
- Concentration panels are declined with surge in homogenous, heterogeneous factors and Schmitt number while augmented with growth in Reynolds number.
- Comparative evaluation of current work demonstrates strong consistency across all findings.
- The effects of various factors on Nusselt numbers and Sherwood numbers have discussed in tabular form.
- The influence of the magnetic factor on the streamline and contour lines patterns of ternary hybrid nanofluid by comparing the cases of (M = 0) and (M = 4) has also explained in detail.

## Data Availability

The data that support the findings of this study are available from the corresponding author upon reasonable request.
